# Infectious salmon anaemia virus (ISAV) in Chilean Atlantic salmon (Salmo salar) aquaculture: emergence of low pathogenic ISAV-HPR0 and re-emergence of virulent ISAV-HPR∆: HPR3 and HPR14

**DOI:** 10.1186/1743-422X-10-344

**Published:** 2013-11-23

**Authors:** Marcos G Godoy, Molly JT Kibenge, Rudy Suarez, Eduardo Lazo, Alejandro Heisinger, Javier Aguinaga, Diego Bravo, Julio Mendoza, Katerina O Llegues, Rubén Avendaño-Herrera, Cristian Vera, Fernando Mardones, Frederick SB Kibenge

**Affiliations:** 1 Centro de Investigaciones Biológicas Aplicadas (CIBA), Diego de Almagro Norte 1013, No. 8, Puerto Montt, Chile; 2 Facultad de Ciencias, Universidad San Sebastián, Lago Panguipulli 1390, Puerto Montt, Chile; 3 ETECMA, Diego de Almagro Norte 1013, No. 10, Puerto Montt, Chile; 4 Department of Pathology and Microbiology, Atlantic Veterinary College, University of Prince Edward Island, 550 University Ave, Charlottetown, P.E.I., C1A 4P3, Canada; 5 Laboratorio de Patología de Organismos Acuáticos y Biotecnología Acuícola, Facultad de Ciencias Biológicas, Universidad Andrés Bello, Viña del Mar, Chile; 6 Interdisciplinary Center for Aquaculture Research (INCAR), Víctor Lamas 1290, PO Box 160-C, Concepción, Chile; 7 Mainstream Chile S.A, Av. Diego Portales 2000, piso 10 y 11, Puerto Montt, Chile; 8 Multiexport Foods, Calle Chorrillos, Puerto Montt 1582, Chile; 9 Center for Animal Disease Modeling and Surveillance (CADMS), Department of Medicine and Epidemiology, School of Veterinary Medicine, University of California, Davis, California 95616, USA

**Keywords:** Low pathogenic infectious salmon anaemia virus, ISAV-HPR0, Virulent ISAV, ISAV-HPR∆, Virulence, *Salmo salar*

## Abstract

**Abstact:**

Infectious salmon anaemia (ISA) is a serious disease of marine-farmed Atlantic salmon (*Salmo salar*) caused by ISA virus (ISAV), which belongs to the genus *Isavirus*, family *Orthomyxoviridae*. ISA is caused by virulent ISAV strains with deletions in a highly polymorphic region (HPR) of the hemagglutinin-esterase (HE) protein (designated virulent ISAV-HPR∆). This study shows the historic dynamics of ISAV-HPR∆ and ISAV-HPR0 in Chile, the genetic relationship among ISAV-HPR0 reported worldwide and between ISAV-HPR0 and ISAV-HPR∆ in Chile, and reports the 2013 ISA outbreak in Chile. The first ISA outbreak in Chile occurred from mid-June 2007 to 2010 and involved the virulent ISAV-HPR7b, which was then replaced by a low pathogenic ISAV-HPR0 variant. We analyzed this variant in 66 laboratory-confirmed ISAV-HPR0 cases in Chile in comparison to virulent ISAV-HPR∆ that caused two new ISA outbreaks in April 2013. Multiple alignment and phylogenetic analysis of HE sequences from all ISAV-HPR0 viruses allowed us to identify three genomic clusters, which correlated with three residue patterns of ISAV-HPR0 (^360^PST^362^, ^360^PAN^362^ and ^360^PAT^362^) in HPR. The virus responsible for the 2013 ISAV-HPR∆ cases in Chile belonged to ISAV-HPR3 and ISAV-HPR14, and in phylogenetic analyses, both clustered with the ISAV-HPR0 found in Chile. The ISAV-HPR14 had the ISAV-HPR0 residue pattern ^360^PAT^362^, which is the only type of ISAV-HPR0 variant found in Chile. This suggested to us that the 2013 ISAV-HPR∆ re-emerged from ISAV-HPR0 that is enzootic in Chilean salmon aquaculture and were not new introductions of virulent ISAV-HPR∆ to Chile. The clinical presentations and diagnostic evidence of the 2013 ISA cases indicated a mixed infection of ISAV with the ectoparasite *Caligus rogercresseyi* and the bacterium *Piscirickettsia salmonis*, which underscores the need for active ISAV surveillance in areas where ISAV-HPR0 is enzootic, to ensure early detection and control of new ISA outbreaks, as it is considered a risk factor. This is the first report of ISA linked directly to the presence of ISAV-HPR0, and provides strong evidence supporting the contention that ISAV-HPR0 shows a strong relationship to virulent ISAV-HPR∆ viruses and the possibility that it could mutate to virulent ISAV-HPR∆.

## Introduction

Infectious salmon anaemia (ISA) is a serious viral disease of marine-farmed Atlantic salmon (*Salmo salar*) caused by ISA virus (ISAV), which belongs to the genus *Isavirus*, family *Orthomyxoviridae*. Mortality in marine fish net-cages rises slowly and can vary from 0 to 90% [1]. In fact, the virus can be present in a net-cage for up to 6 months before significant mortality is noted [2]. This is arguably the most important viral disease of marine-farmed Atlantic salmon (*S, salar*) because of the associated social-economic losses, and ISAV remains an emerging fish pathogen because of the asymptomatic infections in wild and farmed fish and the potential for emergence of new epizootic strains.

ISAV has a segmented genome consisting of eight single-stranded RNA segments [3], in which gene segments 5 and 6 encode the surface glycoproteins that are believed to be important for the pathogenicity of ISAV. The hemagglutinin-esterase (HE), encoded by segment 6, displays both receptor-binding and receptor-destroying enzyme activities [4-6], while gene segment 5 encodes the fusion (F) protein which is responsible for the viral and cellular membranes fusion [7]. The systemic disease ISA is caused by virulent ISAV strains with deletions in a highly polymorphic region (HPR) spanning residues ^337^V to M^372^ in the stem of the HE protein (designated virulent ISAV-HPR∆) that also have either an insertion or the ^266^Q → ^266^L mutation in the F gene [8]. The low pathogenic ISAV (ISAV-HPR0) causes a transient subclinical infection, with replication mainly in Atlantic salmon gills, and because it has the full-length sequence (35 amino acids) of HPR, it is considered to have an ancestral relationship with ISAV-HPR∆ [9]. However, while the deletion or insertion in HPR may be a good genetic marker for differentiation, it is not necessarily the virulence determinant for ISAV. Direct evidence for how the insertion or deletion in HPR affects virulence will only be possible when a reverse genetics system for ISAV is developed.

ISAV-HPR0 viruses have been variously reported in literature (Table 1); they are non-cultivable and detectable only by reverse transcription-polymerase chain reaction (RT-PCR), which causes diagnostic confusion because it can influence the quality of diagnostic test results [10]. ISAV-HPR0 viruses are detected late during ISA outbreaks, and persist long after the disease is contained or eradicated [2,8,11,12]. These viruses deserve special consideration because their evolutionary status is not clearly understood.

**Table 1:** Low pathogenic infectious salmon anemia virus (ISAV-HPR0) detections worldwide

**ISAV-HPR0 designation**	**Country or Region**	**Description**	**Source**
PR European consensus	(*In silico* description)	PR groups; PR variant	Mjaaland *et al.*[13]
HPR0	Scotland	Novel variant of the highly polymorphic region	Cunningham *et al.*[14]
HPR0	North America	HPR0-like variant in North America European-like HPR0 variants	Cook-Versloot *et al.*[15]
HPR0	Norway	ISAV from wild salmon in Scotland, Scot-w	Nylund *et al.*[16]
HPR0	Norway	Non-pathogenic wild type, wild type	Nylund *et al.*[17]
HPR0	Chile	Non-pathogenic viruses	Kibenge *et al.*[8]
HPR0	Norway	Low- or avirulent HPR0 genotype	Markunsen *et al.*[18]
HPR0	Scotland	HPR0 ISAV variant	McBeath *et al.*[11]
HPR0	Iceland	Low/none pathogen ISAv (HPR0) (prevalance in farmed Atlantic salmon stock was approx. 0.6% in 2011 & approx. 0.5% in first half of 2012)	MAST^1^, 2009 (Dr. Marcos Godoy Personal communication)
HPR0	Denmark	HPR0 in 1/9 samples of Atlantic salmon broodstock (August 2010 surveillance sample)	Skall [10]
HPR0	Norway	HPR0 genotypes	Lyngstad *et al.*[19]
HPR0	Faroe Island	Low-pathogenic variant of infectious salmon anemia virus (ISAV-HPR0)	Christiansen *et al.*[12]
SAV-HPR0	Norway	Low virulent infectious salmon anaemia virus (ISAV-HPR0)	Lyngstad *et al.*[20]
HPR0 ISA	Norway	Avirulent (HPR0) viruses HPR0 ISA viruses (avirulent viruses)	Plarre *et al.*[21]

MAST = Matvælastofnun Icelandic Food and Veterinary Authority.

In Chile, the involvement of ISAV in a disease outbreak was officially verified in Atlantic salmon in mid-June 2007 [22] where all isolates that were obtained from outbreaks and had their segment 6 sequenced belonged to ISAV-HPR7b, similar to isolates from Norway, but had acquired a mutation consisting of a 33 base-pair insert in their segment 5 sequence [8]. Phylogenetic analyses of the ISAV isolates from different outbreaks suggested that the virus was introduced from Norway in 1996 [8], probably through fertilized salmon eggs [23,24].

It is now clear that ISAV-HPR0 is enzootic in salmonids populations in Norway, New Brunswick (Canada), Scotland (UK), Faroe Islands, Maine (USA) and Chile [8,9,11,12,20,22,25,26], with the virulent ISAV-HPR∆ replaced by ISAV-HPR0 as the dominant virus variant, but the dynamics of this evolution are still not clearly known [9].

In this study, we describe two major events in the virus-host co-evolution of ISAV in Chilean salmon aquaculture: (1) the nature of emergence and characteristics of ISAV-HPR0 in Chile, and (2) diagnostic findings of the new clinical cases and genetic characterization of the ISAV isolates associated with a brief re-emergence of virulent ISAV-HPR∆ in Chile. Thus we analyzed the laboratory-confirmed ISAV-HPR0 cases in the salmon industry, and the new ISA cases in Chile caused by virulent ISAV-HPR∆ that occurred in April 2013.

## Results and discussion

### Emergence of low pathogenic ISAV (ISAV-HPR0) in Chile

**Figure 1:**
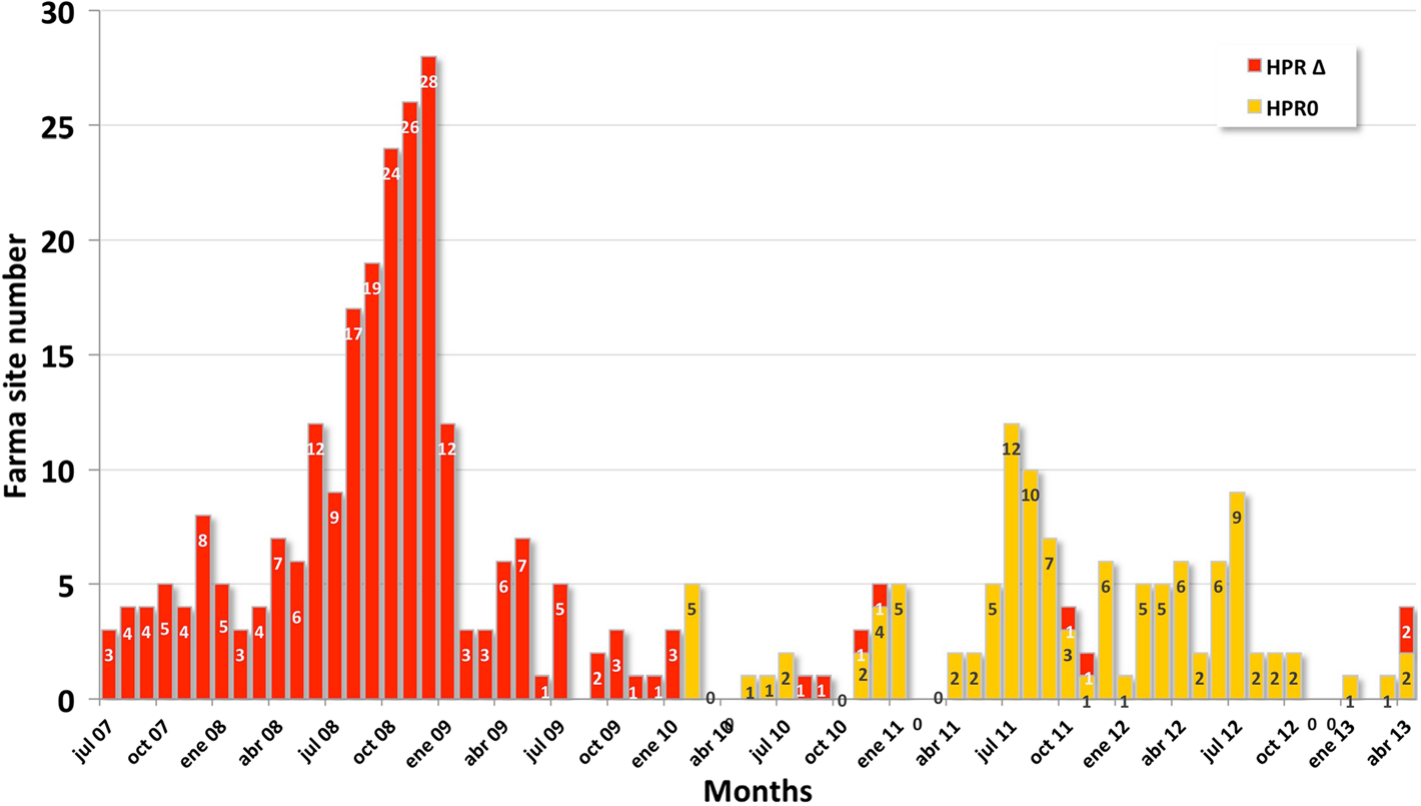
**Number of confirmed infectious salmon anaemia virus (ISAV) cases in Chile from 2007 to 2013.** The red bar corresponds to ISA outbreak (virulent ISAV-HPR∆) and the yellow bar to low pathogenic ISAV (ISAV-HPR0) positive cases.

In Chile, the first clinical case of ISA in marine-farmed Atlantic salmon (*S. salar*) occurred in Chiloe Island (Southern Chile, X Region) in mid-June 2007, and the virus was identified as ISAV HPR7b belonging to the European genotype (or Genotype I) [8,22]. Figure 1 shows the prevalence of laboratory-confirmed ISA and ISAV-HPR0 cases in Atlantic salmon (*S. salar*) farm sites in Chile from July 2007 to April 2013. Following the index case, there was a rapid increase of ISA cases, reaching a peak of 24 cases in November 2008 and then a dramatic drop to December 2010. This decrease was due to a significant decrease in Atlantic salmon biomass being farmed and the implementation of new regulations of biosecurity measures [27].

**Figure 2:**
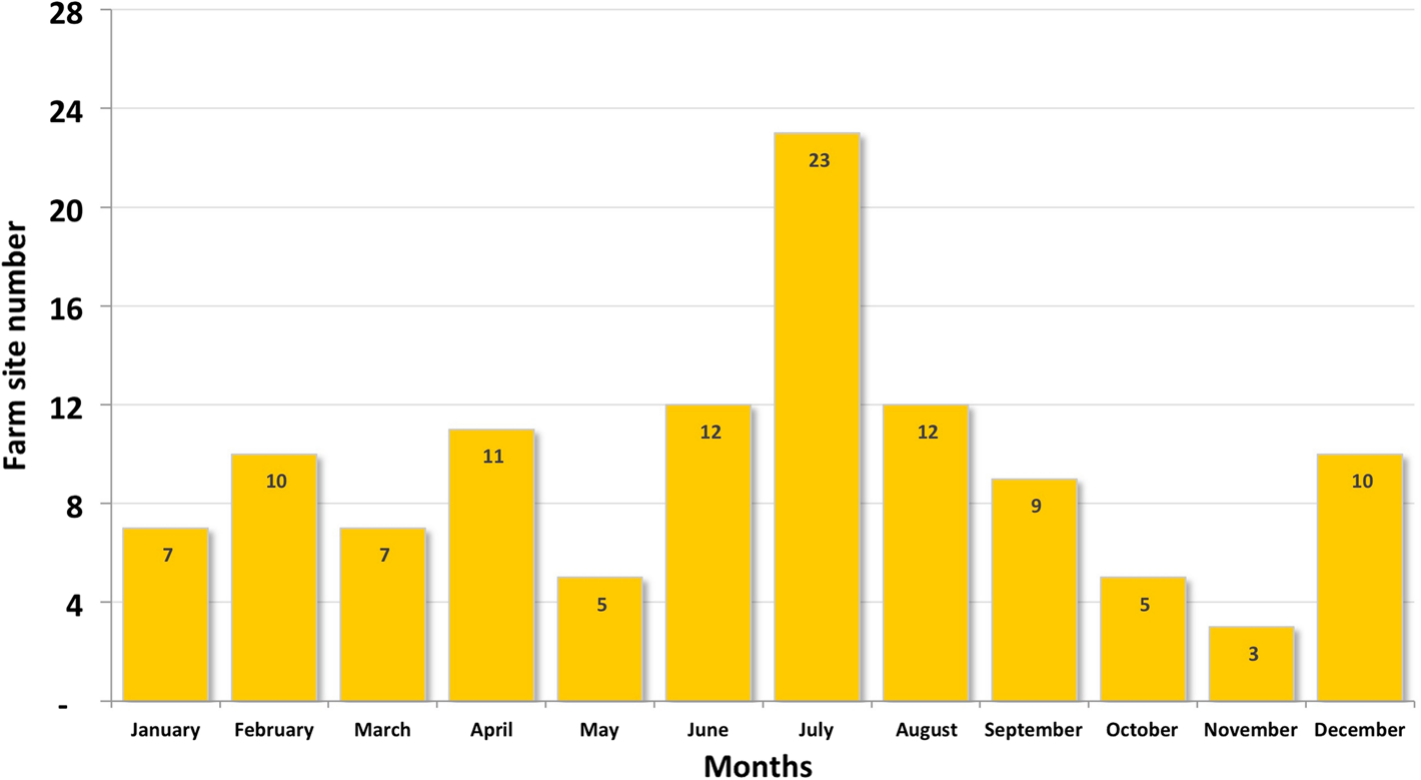
Accumulated monthly frequency of low pathogenic ISAV (ISAV-HPR0) positive farm sites from 2009 to 2013.

The first official record of ISAV-HPR0 was in February 2010. There were a total of 15 laboratory-confirmed ISAV-HPR0 cases in 2010, 53 cases in 2011 and 40 in 2012 (Figure 1). However, prior to these National Fisheries Service (Sernapesca) records, ISAV-HPR0 viruses were detected on three different occasions in 2008 [8], including one at a site in an estuary in the XII Region, i.e. one year after the first clinical case of ISA in Chile [22], and the other two in association with the ISA outbreaks in Chile. Since 2011 there has been a significant increase in ISAV-HPR0 cases in seawater in Chile. Figure 2 shows the seasonality of these ISAV-HPR0 cases, with the largest number (41.2%) occurring in the winter months of June to August. This seasonal pattern of ISAV-HPR0 infection is similar what has been described in Faroe Islands [12], and is suggestive of a transient infection in marine-farmed Atlantic salmon. The replacement of virulent ISAV-HPR∆ by ISAV-HPR0 as the dominant virus variant in Chile is also consistent with the report of ISAV in Faroe Islands [12]. It also suggests that the ancestral relationship between ISAV-HPR0 and ISAV-HPR∆ is a complex one since the origin of ISAV-HPR0 in Chile seems to also confirm the “insertion hypothesis” that the virulent ISAV-HPR∆ undergoes insertion mutations resulting in ISAV-HPR0 and attenuation [28]. The change in the virulence of a pathogen is characterized by a trade-off between transmission success and virulence as defined by host mortality [29-31]. The ISA outbreaks in Chile were related to the sanitary condition in the salmon aquaculture industry [27], and although the virulent ISAV-HPR∆ was replaced by low virulent ISAV-HPR0, the virus remains a risk factor for the industry. Thus, the first virulence wave was related to ISAV-HPR7b in 2008 to 2009 [8], which has been replaced by a low virulence wave related to ISAV-HPR0 infection since 2010, with sporadic virulent outbreaks like the 2013 ISAV-HPR∆ cases. Thus, while the first official detection of HPR0 in Chile was in 2010, Kibenge *et al.*[8] recorded 3 occasions where HPR0 was present in 2008. This indicates that ISAV-HPR0 could have been present much earlier and undetected until biosecurity and surveillance practice improved in the wake of the ISA outbreak [27]. According to official information about ISAV-HPR0 in fresh water, there were 8 cases in 2010, 12 cases in 2011, and 19 cases in 2012, making up 86.2% of a total of 4,356 samples from river, lake and estuary that were reported positive to ISAV-HPR0.

### The cycle threshold (Ct) values of the ISAV-HPR0 cases and 2013 ISAV-HPR∆ cases in Chile show two distinct patterns

A total of 70 cases including 66 laboratory-confirmed ISAV-HPR0 cases and four 2013 ISAV-HPR∆ cases were used in this analysis (Additional file 1: Table S1). The ISAV-HPR0 cases consisted of 1,185 organ pools from 66 fish sites, which were analyzed by real-time reverse transcription-polymerase chain reaction (RT-qPCR) in the period from March 2010 to April 2013. Of these, 323 samples (27.2%) were positive for ISAV-HPR0. The four 2013 ISAV-HPR∆ cases consisted of 57 organ pools from two ISA outbreaks. Of these, 52 samples (91.2%) were positive for ISAV-HPR∆ (Additional file 1: Table S1).

**Figure 3:**
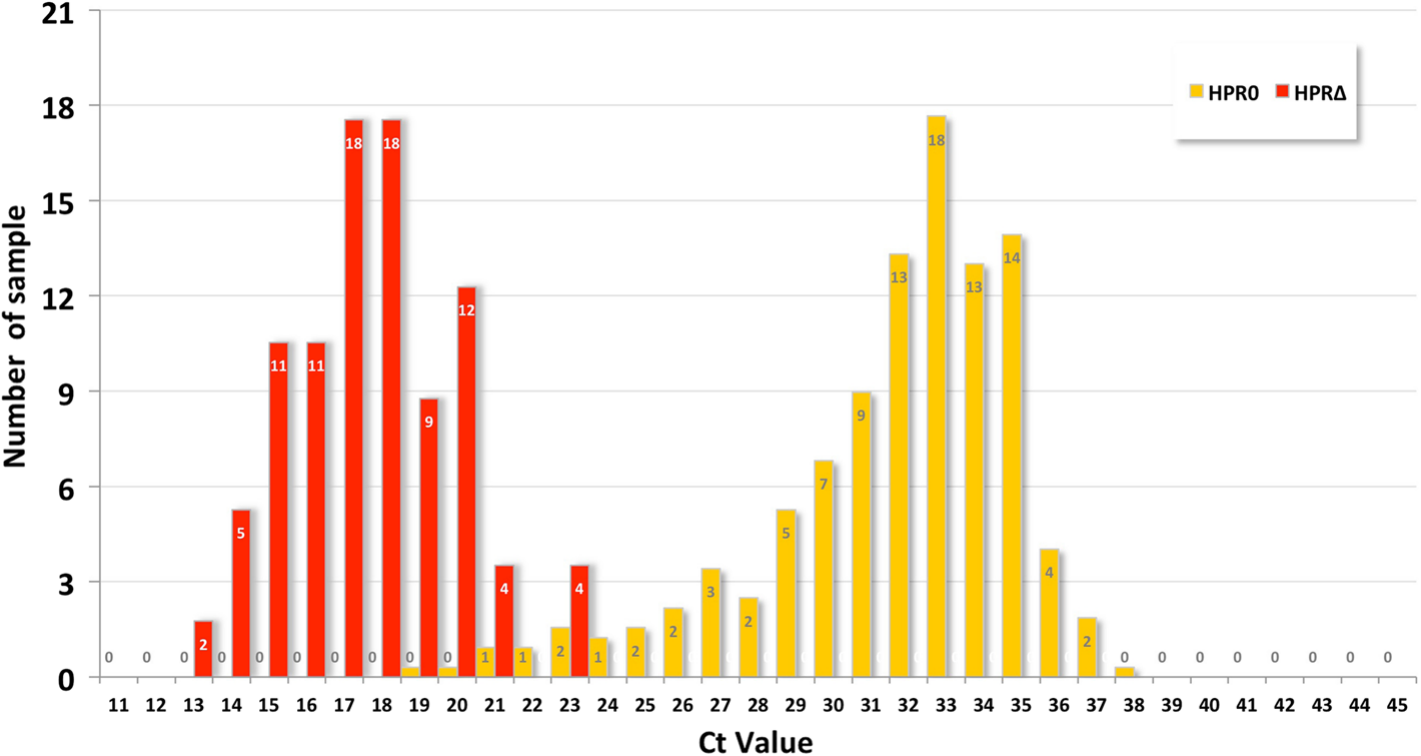
**Frequency distribution of the cycle threshold (Ct) values from all positive samples and all different tissue combinations tested with RT-qPCR for ISAV (listed in Additional file****1:****Table S1).** The yellow bar corresponds to frequency distribution of average Ct values of 66 official ISAV-HPR0 positive farm sites. The red bar corresponds to frequency distribution of four 2013 ISAV-HPR∆ cases.

The Ct values of the ISA-HPR0 positive samples ranged from 19.3 to 38.4, with a mean (± SD) Ct value of 32.3 ± 2.1, whereas the Ct values of the 2013 ISAV-HPR∆ positive samples varied from 13.3 to 34.1 with a mean ± SD Ct value of 18.62 ± 4.5. Figure 3 shows the distribution of average Ct values of 66 ISA-HPR0 cases (yellow), and four ISAV-HPR∆ cases corresponding to the two ISA outbreaks registered in 2013 (red). The ISAV-HPR0 Ct values show a shift to the right which corresponds to high Ct-low pathogenic ISAV, and is in contrast to what we see with the virulent ISAV-HPR∆ where the average Ct shifts to the left which corresponds to low Ct-virulent ISAV. There is a correlation with the virulent ISAV wave in the field where we see low Ct-virulent ISAV and clinical signs followed by the low pathogenic ISAV wave with high Ct-low virulence and no clinical signs. In the virulent ISAV wave we see a systemic infection, whereas in the low pathogenic ISAV wave we see usually a localized gill infection. Thus ISAV infections caused by virulent strains are related to the high pathogen load (low Ct values), with clinical signs and systemic infection and those caused by low pathogenic strains are related to the low pathogen load (high Ct values), without clinical signs and usually external gill infection.

### Spatial and temporal analysis of ISAV-HPR0 cases shows relationship to 2013 ISAV-HPR∆ cases in Chile

**Figure 4:**
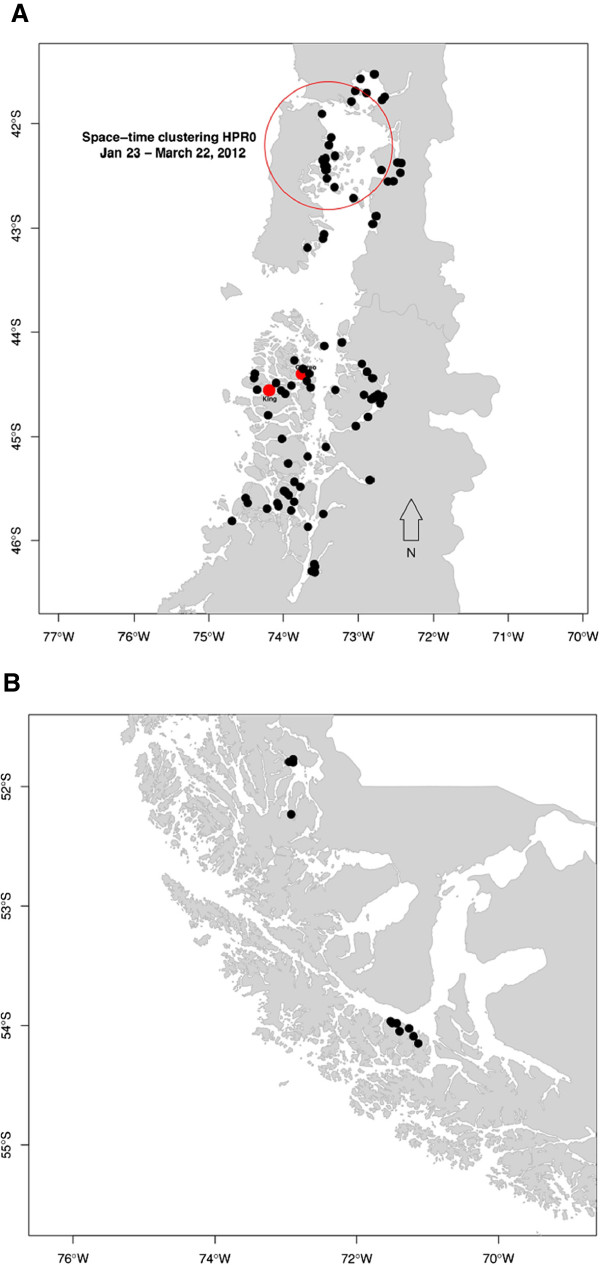
**Geographical distribution of low pathogenic ISAV (ISAV-HPR0) cases and ISA outbreaks from May 2011 to April 2013 in X, XI and XII Regions of Chile. (A)** ISAV-HPR0 and ISA outbreaks in X and XI Regions. The black dots correspond to ISAV-HPR0 positive farm sites. The red dots correspond to the two 2013 ISA outbreaks in XI Region. The red circle indicates the space-time cluster identified from the space-time permutation scan test. **(B)** ISAV-HPR0 in XII Region. The black dots correspond to ISAV-HPR0 positive farm sites.

A total of 93 laboratory-confirmed ISA-HPR0 cases in the period from May 4, 2011, to April 22, 2013, were included in this analysis. Figure 4 shows the geographical distribution of these cases in X and XI Regions (Figure 4A) and XII Region (Figure 4B). The permutation space-time scan statistics detected one significant cluster of ISAV-HPR0 cases (T statistic value = 7.02; P-value = 0.01). This cluster was centered on a farm located at 42.21 South, 73.39 West, with a spatial extension of r = 69.53 km (Figure 4A), and a temporal window from January 23 to March 22, 2012. In the cluster, there were 11 ISAV-HPR0 cases reported, which was 3.83 times more than the expected number of cases (2.87 cases). These results indicate that ISAV-HPR0 infections are somewhat randomly spread. However, the cluster also demonstrates that horizontal transmission could have happened, and not necessarily generating outbreaks. It is possible also that sampling efforts were intensified in that period of time (within one month), but we need more details about that. In XI Region, the two 2013 ISA outbreaks have relationship with the ISAV-HPR0 in 2011 to 2013 (Figure 4A). In Norway, Lyngstad *et al.*[20] previously noted the spatial structure of HPR0 was related to virulent ISA outbreaks.

### Alignment of ISAV segments 5 and 6 sequences reveals presence of only one ISAV-HPR0 in Chile and is related to the 2013 ISAV-HPR∆ (HPR3 and HPR14)

Table 2 shows the multiple alignment of HPR amino acid sequences from selected ISAV-HPR0 reported to date worldwide. The ISAV-HPR0 viruses can be placed into three different groups according to three characteristic residue patterns: ^360^PAN^362^, ^360^PST^362^ and ^360^PAT^362^ in HPR. We consider this to represent three different ISAV-HPR0 subgroups. The first subgroup (^360^PAN^362^) included the majority of the Faroe Islands and the Norway FM173/11 sequences. The second subgroup (^360^PST^362^) included other Faroe Islands, United States and United Kingdom sequences. The third subgroup (^360^PAT^362^) contained all the Norway, Chilean and Sco157/08 (Scotland) sequences (Table 2). This genomic clustering was confirmed by phylogenetic analysis of segment 6 from all ISAV-HPR0 viruses reported to date worldwide as shown in (Additional file 2: Figure S1).

**Table 2:** Alignment of amino acid sequences in the highly polymorphic region (HPR) of the HE gene from selected low pathogenic infectious salmon anaemia virus (ISAV-HPR0) isolates worldwide

**Group**	**ISAV (GenBank Acc. No)**	**Predicted amino acid sequence**^**1**^	**aa deleted**
HPRO	Canada 980712 (AY46060)	TDV-JIRDAIPPQTFNTFNQ**PST**SVLSNIFISM-GVA	0
HPRO	U. K. NWM10 (FJ178189)	TDV-KIRDAIPPQLNQTFNTNQ**PST**SVLSNIFISM-GVA	0
HPRO	USA USA2004 (AY973194)	TDV-KIRVDAIPPQLNQTFNTNQVEQ**PST**SVLSNIFISM-GVA	0
HPRO	Faroe Island FO/03/06 (HQ664993)	TDV-KIRVDAIPPQLNQTFNTNQVEQ**PST**SVLSNIFISM-GVA	0
HPRO	Faroe Island FO/03b/07 (HQ664997)	TDV-KIRVDAIPPQLNQTFNTNQVEQ**PST**SVLSNIFISM-GVA	0
HPRO	Faroe Island FO/08/07 (HQ664998)	TDV-KIRVDAIPPQLNQTFNTNQVEQ**PST**SVLSNIFISM-GVA	0
HPRO	Faroe Island FO/01/06 (HQ664992)	TDV-KIRVDAIPPQLNQTFNTNQVEQ**PAN**SVLSNIFISM-GVA	0
HPRO	Faroe Island FO/01/08 (HQ664999)	TDV-KIRVDAIPPQLNQTFNTNQVEQ**PAN**SVLSNIFISM-GVA	0
HPRO	Faroe Island FO/01a/08 (HQ664994)	TDV-KIRVDAIPPQLNQTFNTNQVEQ**PAN**SVLSNIFISM-GVA	0
HPRO	Faroe Island FO/01b/07 (HQ664995)	TDV-KIRVDAIPPQLNQTFNTNQVEQ**PAN**SVLSNIFISM-GVA	0
HPRO	Scotland 1703/01/86 (AJ440971)	TDV-KIRVDAIPPQLNQTFNTNQVEQ**PAN**SVLSNIFISM-GVA	0
HPRO	Norway FM173/11 (JN711060)	TDV-KIRVDAIPPQLNQTFNTNQVEQ**PAN**SVLSNIFISM-GVA	0
HPRO	Norway H97/04 (DQ108604)	TDV-KIRVDAIPPQLNQTFNTNQVEQ**PAT**SVLSNIFISM-GVA	0
HPRO	Norway H138/08 (JN711063)	TDV-KIRVDAIPPQLNQTFNTNQVEQ**PAT**SVLSNIFISM-GVA	0
HPRO	Chile CGA/1059 (KC414113)	TDV-KIRVDAIPPQLNQTFNTNQVEQ**PAT**SVLSNIFISM-GVA	0
HPRO	Chile CGA/ID758 (KF019742)	TDV-KIRVDAIPPQLNQTFNTNQVEQ**PAT**SVLSNIFISM-GVA	0
HPRO	Scotland Scot157/08 (JN711096)	TDV-KIRVDAIPPQLNQTFNTNQVEQ**PAT**SVLSNIFISM-GVA	0
HPRO	Canada EF6-6 (AY646058)	TDV-KIRVDAIPPQLNQTFNTNQVEQ**PAT**SVLSNIFISM-GVA	0

^1^The characteristic residue patterns representing the three different ISAV-HPR0 subgroups are in **bold**.

Analysis of HPR sequence of the 2013 ISAV-HPR∆ cases revealed that the virus responsible for these outbreaks belonged to ISAV-HPR3 (with 17 amino acid deletions) and ISAV-HPR14 (with 11 amino acid deletions), which are different from the predominant ISAV- HPR∆ (HPR7b with 23 amino acid deletions) associated with the 2007 to 2009 outbreak in Chile [22] (Table 3). The first 2013 ISAV-HPR∆ case was caused by ISAV-HPR3 and the second by ISAV-HPR14, indicating a re-emergence of virulent ISAV-HPR∆. The ISAV-HPR14 had the ISAV-HPR0 residue pattern ^360^PAT^362^, which is the only type of ISAV-HPR0 found in Chile. This suggested to us that the 2013 ISAV-HPR∆ re-emerged from ISAV-HPR0 that is enzootic in Chilean salmon aquaculture and were not new introductions of virulent ISAV-HPR∆ to Chile. ISAV-HPR3 and ISAV-HPR14 were previously detected in isolated cases in X and XI Regions in Chile during the 2007 to 2009 outbreak [8,32].

**Table 3:** Alignment of amino acid sequences in the highly polymorphic region (HPR) of the HE genes from selected low pathogenic infectious salmon anaemia virus (ISAV-HPR0) and virulent infectious salmon anaemia virus (ISAV-HPR∆)

**Group**	**ISAV isolate (GenBank Acc. No.)**	**Predicted amino acid sequence**^**1**^																																									**aa deleted**
HPRO	United Kingdom NWM10 (FJ178189)	T	D	V	K	I	R	V	D	A	I	P	P	Q	L	N	Q	T	F	N	T	N	Q	V	E	Q	**P**	**S**	**T**	S	V	L	S	N	I	F	I	S	M	G	V	A	0
HPRO	Scotland 1703/01/86 (AJ440971)	T	D	V	K	I	R	V	D	A	I	P	P	Q	L	N	Q	T	F	N	T	N	Q	V	E	Q	**P**	**A**	**N**	S	V	L	S	N	I	F	I	S	M	G	V	A	0
HPRO	Chile CH29/o8 (JN711094)	T	D	V	K	I	R	V	D	A	I	P	P	Q	L	N	Q	T	F	N	T	N	Q	V	E	Q	**P**	**A**	**T**	S	V	L	S	N	I	F	I	S	M	G	V	A	0
HPR20	Canada RPC/NB 98-0280-2 (AF294870)	T	D	V	K	I	R	V	D	A	I	-	-	-	L	-	-	-	-	G	V	N	Q	V	E	Q	**P**	**S**	**T**	S	V	L	S	N	I	F	I	S	M	G	V	A	7
HPR36	Norway Vir22 (DQ785258)	T	D	V	K	I	R	V	D	A	-	-	-	-	-	-	-	-	F	N	T	N	Q	V	E	Q	**P**	**A**	**T**	S	V	L	S	N	I	F	I	S	M	G	V	A	8
HPR6	Norway MR60/01 (AY127876)	T	D	V	K	I	R	V	D	A	I	-	-	-	-	-	-	-	-	-	-	-	Q	V	E	Q	**P**	**A**	**T**	S	V	L	S	N	I	F	I	S	M	G	V	A	11
HPR14	Norway ST21/96 (AF364886)	T	D	V	K	I	R	V	D	A	-	-	-	-	-	-	-	-	-	-	-	N	Q	V	E	Q	**P**	**A**	**T**	S	V	L	S	N	I	F	I	S	M	G	V	A	11
HPR33	Norway N127/07 (JN711066)	T	D	V	K	I	R	V	D	-	-	-	-	-	-	-	-	-	-	-	D	N	-	V	E	Q	**P**	**A**	**T**	S	V	L	S	N	I	F	I	S	M	G	V	A	12
HPR17	Norway MR103/05 (DQ108606)	T	D	V	K	I	R	-	-	-	-	-	-	-	L	-	-	-	-	-	-	-	E	V	E	Q	**P**	**A**	**T**	S	V	L	S	N	I	F	I	S	M	G	V	A	14
HPR9b	Chile 2006B-13364 (FJ594284)	T	D	V	K	I	R	V	D	A	I	P	P	Q	L	N	Q	T	F	N	T	-	-	-	-	-	-	-	-	-	-	-	-	-	-	-	I	S	M	G	V	A	15
HPR12	Norway N5/89 (AY127882)	T	D	V	K	I	R	V	D	A	I	P	P	Q	L	N	Q	T	-	-	-	-	-	-	-	-	-	-	-	-	-	-	-	N	I	F	I	S	M	G	V	A	15
HPR21	Chile 7833–1 (AF294879)	T	D	V	K	I	R	V	D	A	I	P	P	Q	L	-	-	-	-	-	-	-	-	-	-	-	-	-	-	-	-	-	S	N	I	F	I	S	M	G	V	A	17
HPR3	Norway ST110/05 (DQ108598)	T	D	V	K	I	R	V	D	A	I	P	P	Q	L	N	Q	T	-	-	-	-	-	-	-	-	-	-	-	-	-	-	-	-	-	F	I	S	M	G	V	A	17
HPR4	Norway Gullesfjord/94 (AF302801)	T	D	V	K	I	R	V	D	A	I	P	P	Q	L	-	-	-	-	-	-	-	-	-	-	-	-	-	-	-	-	-	S	N	I	F	I	S	M	G	V	A	17
HPR4c	Chile 26560–10 (EU625666)	T	D	V	K	I	R	V	D	A	I	P	P	Q	L	-	-	-	-	-	-	-	-	-	-	-	-	-	-	-	-	-	-	N	I	F	I	S	M	G	V	A	18
HPR9	Norway FM116/06 (JN711058)	T	D	V	K	I	R	V	D	A	I	P	P	Q	L	N	Q	T	F	N	T	-	-	-	-	-	-	-	-	-	-	-	-	-	-	-	-	-	-	G	V	A	18
HPR10	Norway MR52/00 (AF364892)	T	D	V	K	I	R	-	-	-	-	-	-	-	-	-	-	-	-	-	-	-	-	-	-	Q	**P**	**A**	**T**	S	V	L	S	N	I	F	I	S	M	G	V	A	18
HPR12a	Norway ISAV3 (DQ785247)	T	D	V	K	I	R	V	D	A	I	P	P	Q	L	-	-	-	-	-	-	-	-	-	-	-	-	-	-	-	-	-	-	N	I	F	I	S	M	G	V	A	18
HPR31	Norway T126/07 (JN711076)	T	D	V	K	I	R	V	D	A	I	P	P	Q	L	N	-	-	-	-	-	-	-	-	-	-	-	-	-	-	-	-	-	-	I	F	I	S	M	G	V	A	18
HPR11	Norway SF57/00 (AF364890)	T	D	V	K	I	R	V	D	A	I	P	P	-	-	-	-	-	-	-	-	-	-	-	-	-	-	-	-	-	-	-	R	N	I	F	I	S	M	G	V	A	19
HPR13	Faroe Island 1173/01/12 (AJ440970)	T	D	V	K	-	-	-	-	-	-	-	-	-	-	-	-	-	-	-	-	-	-	-	E	Q	**P**	**A**	**N**	S	V	L	S	N	I	F	I	S	M	G	V	A	19
HPR16	Norway T90/04 (AY971666)	T	D	V	K	I	R	V	D	A	I	P	P	Q	L	N	Q	T	F	-	-	-	-	-	-	-	-	-	-	-	-	-	-	-	-	-	-	-	M	G	V	A	19
HPR21b	Canada NB1330-2 (AY646063)	T	D	V	K	I	R	V	D	A	I	P	P	Q	L	N	Q	T	-	-	-	-	-	-	-	-	-	-	-	-	-	-	-	-	-	-	-	-	M	G	V	A	20
HPR35	Norway NT156/09 (JN711072)	T	D	V	K	I	R	V	D	A	I	P	P	Q	L	N	Q	T	F	-	-	-	-	-	-	-	-	-	-	-	-	-	-	-	-	-	-	-	-	G	V	A	20
HPR2	Norway T131/07 (JN711078)	T	D	V	K	I	R	V	D	A	I	P	P	Q	L	N	Q	T	-	-	-	-	-	-	-	-	-	-	-	-	-	-	-	-	-	-	-	-	M	G	V	A	20
HPR19	Norway SF18/96 (AF364869)	T	D	V	K	I	R	V	D	A	I	P	P	Q	L	N	Q	T	-	-	-	-	-	-	-	-	-	-	-	-	-	L	-	-	-	-	-	-	-	G	V	A	20
HPR1	Norway FM86/04 (AY971659)	T	D	V	K	-	-	-	-	-	-	-	-	-	-	-	-	-	-	-	-	-	-	-	-	-	**P**	**A**	**T**	S	V	L	S	N	I	F	I	S	M	G	V	A	21
HPR5	Norway T152/09 (JN711086)	T	D	V	K	I	R	V	D	A	I	P	P	Q	L	-	-	-	-	-	-	-	-	-	-	-	-	-	-	-	-	-	-	-	-	-	I	S	M	G	V	A	21
HPR34	Norway NT134/08 (JN711069)	T	-	-	-	-	-	-	-	-	-	-	-	-	-	-	-	-	-	-	-	-	-	-	-	Q	**P**	**A**	**T**	S	V	L	S	N	I	F	I	S	M	G	V	A	21
HPR1c	Chile 31606-H (FJ594281)	T	D	V	-	-	-	-	-	-	-	-	-	-	-	-	-	-	-	-	-	-	-	-	-	-	-	A	T	S	V	L	S	N	I	F	I	S	M	G	V	A	23
HPR7b	Chile 31991-3N (FJ786983)	T	D	V	K	-	-	-	-	-	-	-	-	-	-	-	-	-	-	-	-	-	-	-	-	-	-	-	T	S	V	L	S	N	I	F	I	S	M	G	V	A	23
HPR15	Norway H56/00 (AF364880)	T	D	V	-	-	-	-	-	-	-	-	-	-	-	-	-	-	-	-	-	-	-	-	E	-	-	-	T	S	V	L	S	N	I	F	I	S	M	G	V	A	23
HPR15c	Chile 901 (GU830908)	T	D	V	-	-	-	-	-	-	-	-	-	-	-	-	-	-	-	-	-	-	-	-	-	-	-	A	T	S	V	L	S	N	I	F	I	S	M	G	V	A	23
HPR15e	32246 (FJ86988)	T	D	V	-	-	-	-	-	-	-	-	-	-	-	-	-	-	-	-	T	-	-	-	-	-	-	-	T	S	V	L	S	N	I	F	I	S	M	G	V	A	23
HPR8	Norway MR46/99 (AF364896)	T	D	V	K	I	R	V	D	A	I	P	P	Q	L	-	-	-	-	-	-	-	-	-	-	-	-	-	-	-	-	-	-	-	-	-	-	-	-	G	V	A	24
HPR15b	cHILE 31150–3 (FJ786985)	T	D	V	-	-	-	-	-	-	-	-	-	-	-	-	-	-	-	-	-	-	-	-	-	-	-	-	T	S	V	L	S	N	I	F	I	S	M	G	V	A	24

^1^The characteristic residue patterns representing the three different ISAV-HPR0 subgroups are in **bold**.

Additional file 3: Table S4 shows a multiple alignment of amino acid sequences in the proteolytic cleavage site of the Fusion glycoprotein of selected ISAV isolates from the GenBank and the segment 5 sequence of the 2013 ISAV-HPR∆ cases. Both the 2013 ISAV-HPR3 and ISAV-HPR14 isolates have a L at position 266 and no insert sequence; the F protein of ISAV-HPR0 found in Chile has a Q at position 266 and no insert sequence [8]. In contrast, the ISAV-HPR∆ from the 2007 to 2009 outbreak were characterized by the dominance of ISAV-HPR7b, which have the F protein with a Q at position 266 and an 11-amino acid insert [8].

### Phylogenetic analyses of ISAV HE and F glycoprotein genes show ISAV-HPR0 and 2013 ISAV-HPR∆ (HPR3 and HPR14) in Chile form a distinct cluster

**Figure 5:**
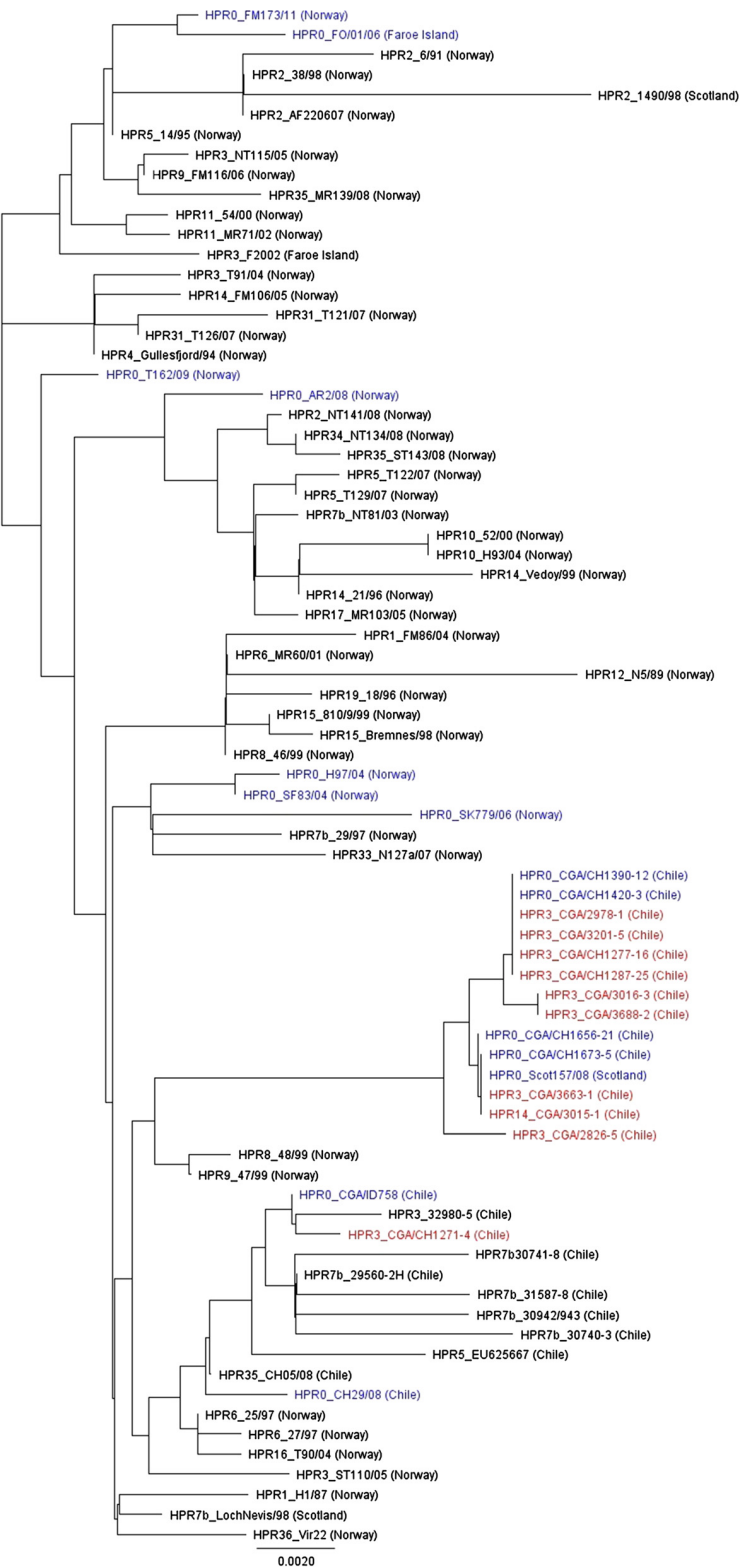
**Phylogenetic tree of segment 6 sequences from selected ISAV isolates of European genotype.** The analysis was performed using 1008 nucleotides of the 5′ portion of the HE gene (excluding the HPR). The phylogenetic tree was constructed by maximum likelihood (ML) using Tamura-Nei and Neighbor-joining [51]. All ISAV-HPR0 viruses are shown in blue and the ISAV-HPR∆ viruses (ISAV-HPR3 and ISAV-HPR14) associated with the 2013 ISA outbreaks in Chile are shown in red.

The phylogenetic analysis of segment 6 sequences from selected ISAV isolates of European genotype, including sequences from ISAV-HPR0 and ISAV-HPR∆ viruses in Chile is presented in Figure 5. The Chilean viruses form a distinct cluster from all the other isolates from Norway, Faroe Islands, Scotland/UK, Canada, and USA, except for HPR8_48/99, HPR9_47/99, HPR6_25/97, HPR6_27/97, HPR16_T90/04, and HPR0_Scot157/08 from Scotland, which cluster with the 2013 ISAV-HPR∆ viruses in Chile. Interestingly, the 2013 ISAV-HPR∆ viruses (ISAV-HPR3_CGA/2978, CGA/3201, CGA/CH1277, CGA/CH1287, CGA/3016, CGA/3663, CGA/3688, ISAV-HPR14_CGA/3015, and ISAV-HPR0_CGA/CH1390, CGA/CH1420, CGA/CH1656, CGA/CH1673) form a tight group with high genetic similarity (99.9% identity) separate from the ISAV-HPR∆ viruses previously reported in Chile (Figure S2). However, the ISAV-HPR3_CGA/CH1271 isolate responsible for the first ISA outbreak in 2013 and the 2012 reported ISAV-HPR0 (ISAV-HPR0_CGA/ID758) had high genetic similarity to ISAV-HPR3_32980-5 from 2008 [8]. There are no previous sequences for ISAV-HPR14 in Chile, but this virus was first detected in 2008 in XI Region [32]. The 2013 ISAV-HPR∆ viruses (ISAV-HPR3 and ISAV-HPR14) show very close similarity to the ISAV-HPR0_CGA/CH1390, CGA/CH1420, CGA/CH1656, and CGA/CH1673, (this study) and ISAV-HPR0_CH29/08 [21] (Figure 5, and Additional file 4: Figure S2). The relationship between ISAV-HPR0 and ISAV-HPR∆ has been previously described in Norway [18-21] and Faroe Islands [12], but this is the first report of ISA cases directly linked to the presence of ISAV-HPR0 that is enzootic in an area.

**Figure 6:**
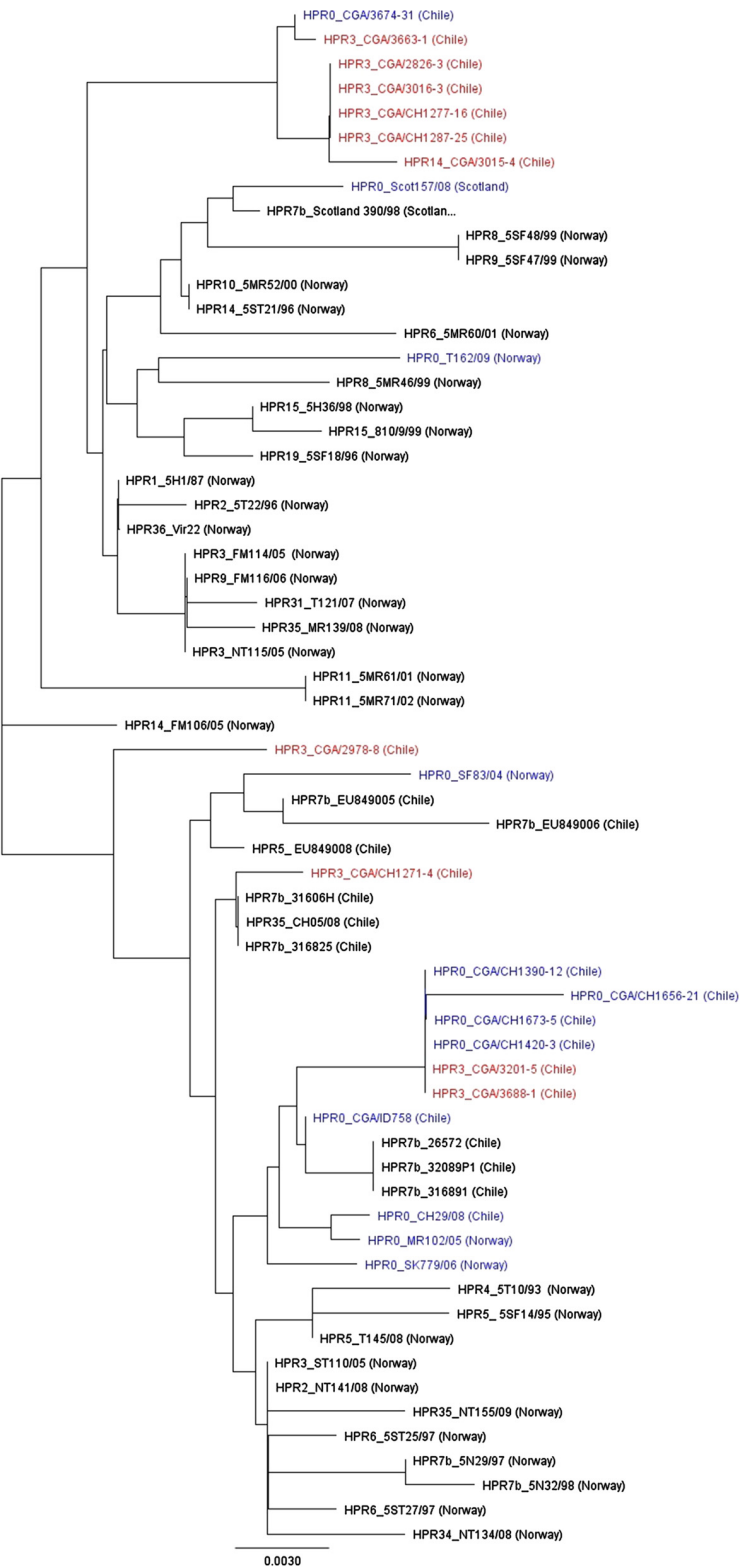
**Phylogenetic tree of segment 5 sequences from selected ISAV isolates of European genotype.** The analysis was performed using 625 nucleotides of the 5′ portion of the F gene (excluding the nucleotides responsible for the Q266L mutation). The phylogenetic tree was constructed by maximum likelihood (ML) using Tamura-Nei and Neighbor-joining [51]. All ISAV-HPR0 viruses are shown in blue and the ISAV-HPR∆ viruses (ISAV-HPR3 and ISAV-HPR14) associated with the 2013 ISA outbreaks in Chile are shown in red.

The phylogenetic analysis of segment 5 sequences from selected ISAV isolates of European genotype, including sequences from ISAV-HPR0 and ISAV-HPR∆ viruses in Chile is presented in Figure 6. Similarly to segment 6 sequences (Figure 5), the Chilean 2013 ISAV-HPR∆ viruses form two main subgroups; a major subgroup (ISAV-HPR3_CGA/2826, CGA/CH1277, CGA/CH1287, CGA/3016, CGA/3663, and ISAV-HPR14_CGA/3015, is very closely related to Chilean ISAV-HPR0_CGA/CH3674 and a minor subgroup (ISAV-HPR3_CGA/CH1271, CGA/CH3201 and CGA/CH3688) is very closely related to Chilean ISAV-HPR0_CGA/CH1390, CGA/CH1420, CGA/CH1656, and CGA/CH1673 (Figure 6). It is noteworthy that the ISAV-HPR0_Scot157/08 from Scotland which grouped with the 2013 ISAV-HPR∆ viruses in Chile on segment 6 is not related to these viruses on segment 5 (Figure 6).

### The clinical presentations of the ISA cases of 2013 were the result of three Atlantic salmon (*Salmo salar*) pathogens

**Figure 7:**
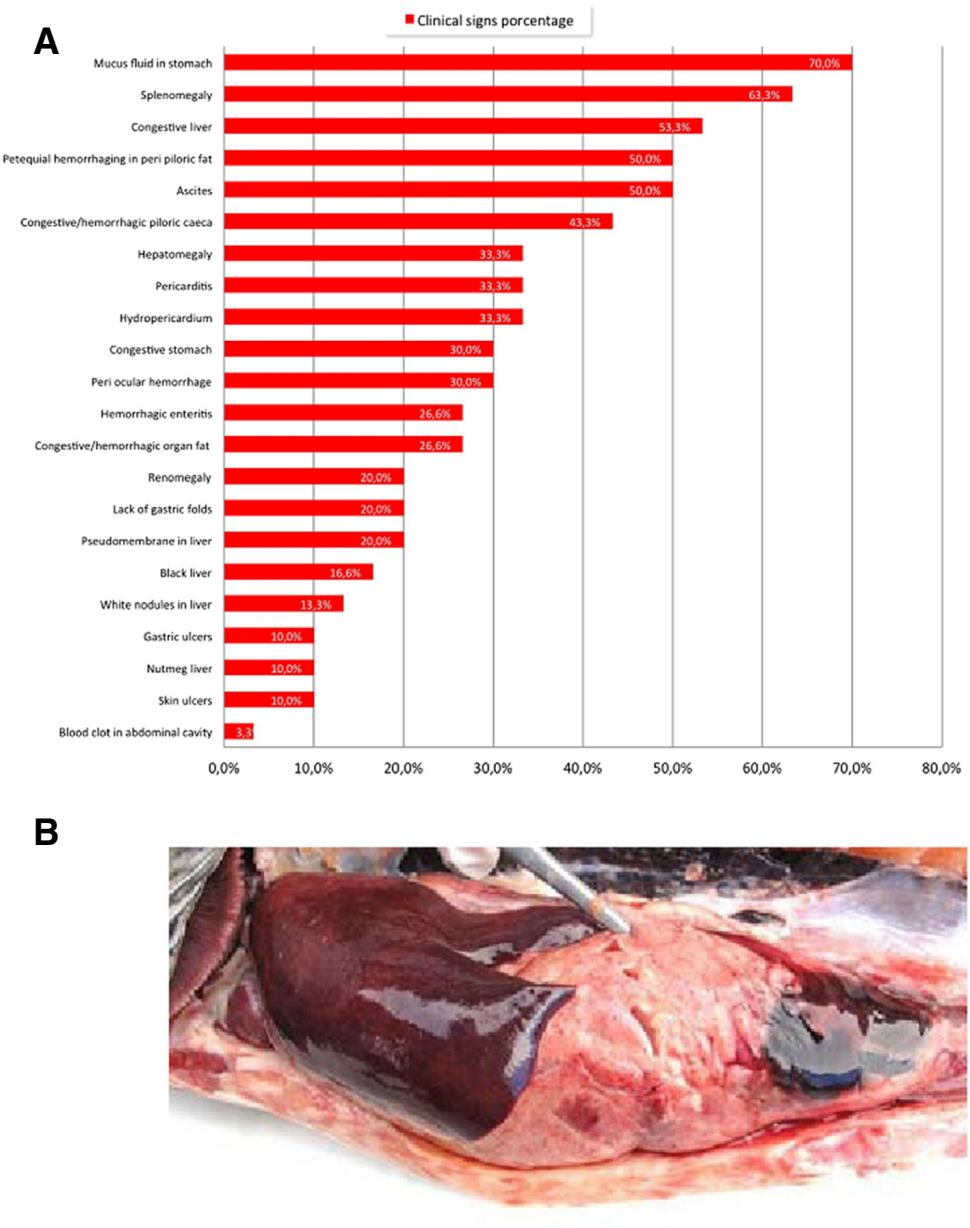
**Clinical presentation of Atlantic salmon (*Salmo salar*) from the 2013 ISA outbreak. (A)** Frequency of the external and internal gross lesions in affected Atlantic salmon from the 2013 ISA outbreak. Percentage of fish with a specified lesion among 38 fish necropsied. **(B)** Gross lesions at necropsy: Atlantic salmon with very dark liver and congestion and hemorrhages on the visceral adipose tissue.

In the first new ISA case, which was diagnosed on April 4, 2013, the fish were affected by a parasite (*Caligus rogercresseyi*), a bacterium (*Piscirickettsia salmonis*) and a virus (ISAV). The pathology findings were the result of these three pathogens in the fish. The affected fish were lethargic, moribund, near to the net and some of them with abnormal swimming behaviour (Additional file 5: Figure S3). Figure 7A summarizes the frequency of the external and internal gross lesions noted from 30 affected fish at necropsy. The most frequent external lesion was hemorrhages in the eye (30%) and petechial hemorrhaging on the skin related mainly to *C. rogercresseyi* infection. Internally, the most frequent gross lesions were hydropericardium and liver congestion (33.3% each). Hepatomegally, splenomegally and liver pseudomembranes are classical lesions of Rickettsial salmonid septicemia (SRS). Liver congestion, black liver (Figure 7B), gastric congestion, peri-pyloric fat petechial hemorrhaging, and congestion in pyloric ceca are clinical presentations of ISA. The gross pathology is explained by a mixed infection of Caligidosis, SRS, and ISA. The histopathology findings were characteristic of ISA and SRS (Additional file 6: Figure S4). These morphological changes related to ISA infection are similar to those described by Evensen *et al.*[33], Spielberg *et al.*[34], Simko *et al.*[35], Jones and Groman [36], Moneke *et al.*[37], and Godoy *et al.*[22].

The fish tissue samples were positive for ISAV, PRV and *P. salmonis* and negative for VHSV, EHNV, IHNV, and SAV by RT-PCR testing. Homogenates of heart, spleen, and kidney tissues from the 2013 ISA outbreaks (ISAV-HPR3 and ISAV-HPR-14) were inoculated on monolayers of Atlantic salmon Kidney (ASK1) and Salmon head kidney (SHK-1); the ISAV-HPR3 case produced cytopathic effects (CPE) in ASK1 and SHK-1 after 10 and 12 days post inoculation during primary isolation, respectively, whereas the ISAV-HPR14 case produced CPE in ASK1 and SHK-1 after 14 and 10 days post inoculation during primary isolation, respectively (data not shown). The presence of ISAV in the cell lysates was confirmed using RT-PCR. Neither CPE nor presence of ISAV was detected by RT-PCR in inoculated cultures of Chinook salmon embryo (CHSE-214) and Bluegill Fry (BF2) cell lines.

### Concluding remarks

Our work shows that by 2012, the low pathogenic ISAV-HPR0 had completely replaced the virulent ISAV-HPR∆ responsible for the 2007–2010 ISA outbreaks as the dominant virus variant in marine-farmed Atlantic salmon in Chile. The occurrence of two new ISA outbreaks in April 2013 marked a brief re-emergence of virulent ISAV-HPR∆, and genetic analysis of the ISAV isolates strongly suggests they were not new introductions of virulent ISAV-HPR∆ to Chile. This is the first report of ISA cases linked to the presence of ISAV-HPR0 that is enzootic in an area, and provides strong evidence supporting the contention that given the right conditions, ISAV-HPR0 can mutate to virulent ISAV-HPR∆ viruses. The mixed clinical presentation involving caligidosis, SRS, and ISA in the 2013 ISA outbreaks underscores the need for active ISAV surveillance in areas where ISAV-HPR0 is enzootic, to ensure early detection and control of new ISA outbreaks, as it is considered a risk factor.

## Materials and methods

### Study material

This research investigated two recent major events in the virus-host co-evolution of ISAV in Chilean salmon aquaculture between 2009 and 2013: (1) the diagnostic and molecular characteristics of the emergence of low pathogenic infectious salmon anemia virus (ISAV-HPR0) that replaced the original virulent infectious salmon anemia virus (ISAV-HPR∆) as the dominant virus variant in Chile, and (2) the new ISA cases and molecular characterization of the ISAV isolates associated with a brief re-emergence of virulent ISAV-HPR∆ in Chile in April 2013. The emergence of low pathogenic ISAV-HPR0 and the 2013 re-emergence of virulent ISAV-HPR∆ were analyzed from 3 datasets: (a) the official data of ISA cases provided in the specific surveillance program for ISA in Chile between 2009 and 2013 provided by Sernapesca, (b) the data provided by laboratories about the nature and characteristics of diagnosis of emerging ISAV-HPR0 and re-emergence of virulent ISAV-HPR∆, and (c) molecular characterization of ISAV segments 5 and 6 from positive ISAV cases confirmed by ETECMA laboratory.

### Investigation of the emergence of ISAV-HPR0 cases in Chile

In order to determine the evolution of the ISAV-HPR0 positive cases and ISA confirmed outbreaks in Chile, a request was made to Sernapesca according to the public statute accession information number 20.285, for the official data reported from 2009 to 2012. A new request was also made for the official data on ISAV-HPR0 reported from 2009 to 2012 in fresh water. The data provided by Sernapesca included the monthly frequency and geographical distribution of the ISAV-HPR0 and ISAV-HPR∆ positive cases.

### Field sampling of ISA cases of 2013

Atlantic salmon *(Salmo salar*) moribund fish held in seawater rearing cages from affected farm sites were necropsied and the main clinical signs were registered. Samples were collected and submitted to ETECMA Laboratory, Chile, for histopathology and virus detection by real-time reverse transcription-polymerase chain reaction (RT-qPCR) and cell culture.

### Gross pathology of ISA cases of 2013

The significant external and internal gross lesions were registered and the frequency of each of these was determined and plotted.

### Histological analysis of ISA cases of 2013

Liver, spleen, kidney, stomach, heart, pyloric caeca and intestinal tissue samples for histological analysis were collected in 10% buffered formalin. They were then processed using standard procedures and the sections were stained with Haematoxylin & Eosin (H&E), in order to describe the significant morphological changes.

### Virus isolation from ISA cases 2013

Tissue samples for virus isolation attempts were collected in Minimal Essential Medium (MEM) (GIBCO) and were shipped cold to the laboratory. Homogenized heart, spleen, and kidney tissues were inoculated on monolayers of Chinook salmon embryo (CHSE-214), Atlantic salmon Kidney (ASK1), Atlantic salmon head kidney (SHK-1) and Bluegill Fry (BF-2) cell lines following standard protocols in the OIE Aquatic Manual [1]. Briefly, each tissue was weighed and macerated to a 10% homogenate w/v in PBS with 10x antibiotics. The homogenates were centrifuged at 3,000 rpm for 15 min at 4°C. The supernatants were individually filtered using 0.45 μM syringe filters to remove any bacteria prior to use in virus isolation attempts. 24 hr-old cell monolayers in tissue culture flasks free of medium were inoculated with the sample supernatant diluted 1:10 in serum-free medium, and incubated for 2 hr at room temperature to allow for virus adsorption. Maintenance medium was then added and the inoculated cells were then incubated at 16°C and infection was allowed to proceed with daily monitoring using an inverted light microscope until the CPE was evident or 21 days and the flasks were frozen at −80°C. Virus isolation was monitored by RT-PCR on the cell lysates since virus replication may occur without development of apparent CPE [38]. CPE negative and RT-PCR negative cultures were passaged on fresh cell monolayers. A sample was considered negative if no CPE or positive RT-PCR was observed after three blind passages.

### Laboratory investigations of ISAV-HPR0 and ISAV-HPR∆ cases

For the diagnostic characterization of ISAV-HPR0 and ISAV-HPR∆ cases, a full necropsy was conducted on the subject fish, and pooled tissue samples (gills, heart and head kidney) were collected for RT-qPCR analysis. For the 2013 clinical cases, the kidney, heart, gill, and liver tissues were collected individually directly in ethanol 70% (v/v) or RNALater® (Ambion Inc). Total RNA was extracted using the protocol of each diagnostic laboratory according to the standard protocol by Sernapesca for the detection of ISAV in tissue homogenates. ISAV primers and specific conditions used were as described by Snow *et al.*[39] targeting ISAV segment 8. Samples were considered ISAV positive based on cycle threshold (Ct) values according to the laboratory procedure. The confirmation of ISAV-HPR0 cases was made by sequence analysis of RT-PCR products obtained using segment 6 HPR primers in accordance with previously described methods [8,14,19] and/or by specific detection of ISAV-HPR0 by RT-qPCR with TaqMan® chemistry and ISAV-HPR0 TaqMan® probe according to a proprietary ETECMA protocol. To ensure efficient performance of each assay, a constitutively expressed endogenous gene, eukaryotic elongation factor 1-alpha (ELF1α), was used as an internal control reference gene [39].

For the 2013 clinical cases investigated by the ETECMA Laboratory, automated tissue homogenization was performed using the MagNA Lyser instrument (Roche). Total RNA was extracted using a robot (Roche MagNA Pure LC instrument with the MagNA Pure LC RNA isolation kit III - Tissue (for virus) and Isolation kit III – Tissue (for bacteria and fungi), according to the manufacturer's instructions. The RT-qPCR analysis was made using LightCycler 480 RNA Master Hydrolysis Probes for RNA and LightCycler 480 Probe Master). Fish tissue samples were also tested for exotic and enzootic pathogens such as piscine reovirus (PRV) using methods described by Palacios *et al.*[40], viral hemorrhagic septicemia virus (VHSV) using methods described by Garver *et al.*[41], epizootic hematopoietic necrosis virus (EHNV) and infectious hematopoietic necrosis virus (IHNV) using methods described by Holopainen *et al.*[42], salmon alphavirus (SAV) using methods described by Hodneland *et al.*[43] and *P. salmonis* using methods described by Corbeil *et al.*[44], with minor modifications.

### Identification of temporal-spatial clusters of ISAV-HPR0 cases

Clusters of ISAV-HPR0 cases in a particular area and during the specific period of time were identified by space-time permutation scan statistics [45]. Briefly, the space-time permutation approach centred a hypothetical cylinder at the geospatial coordinates of each location where ISAV-HPR0 cases were reported. The base and height of the cylinder, representing space and time, respectively, vary up to a maximum value that determines the possible maximum size of the cluster. The ratio between observed and expected number of cases within each candidate cylinder is computed and the significance of the cluster is tested using a Monte Carlo simulation approach [46]. Briefly, a large number (say, 999) of simulated datasets are generated by randomizing the days d when the cases were observed and assigning them to the original set of locations in 999 consecutive iterations. The likelihood of the candidate cluster is computed for each simulated dataset and the proportion of times in which the observed likelihood ratio is lower than the likelihood ratios estimated for the 999 simulated datasets is considered a proxy for the probability that the cluster occurs by chance (P-value). Candidate clusters with a computed P < 0.05 were assumed to represent clusters of HPR0 cases. Cluster analyses were performed using the SaTScan software version 9.1.1 [47], and results were mapped using the software R [48].

### Molecular characterization of ISAV segments 5 and 6 from of ISAV-HPR0 and ISAV-HPR∆ cases

For this study, the segment 6 HPR of ten ISAV-HPR0 cases and all positive samples having Ct values below 34 from the 2013 ISAV-HPR∆ cases were amplified using the primer set published by Kibenge, *et al*. [8]. The full length segments 5 and 6 of ISAV-HPR0 cases and samples from the two 2013 ISAV-HPR∆ were amplified using the primers published by McBeath *et al.*[11]. Conventional RT-PCR was carried out using the EXPRESS qPCR SuperMix with Premixed ROX (Invitrogen), in a Swift^TM^ MaxPro Thermal Cycler (Esco Healthcare Pte. Ltd.). The PCR products were then purified by agarose gel electrophoresis and directly sequenced. DNA sequencing was performed by Macrogen Inc. (South Korea) The sequences were analyzed with Genius v 6.0.6 software [49] and subjected to a BLAST search [50] against the latest release at GenBank.

The evolutionary distance of ISAV-HPR0 HE gene sequenced in this study, together with the reference sequences deposited in GenBank was estimated with neighbor-joining phylogenetic analysis [51]. A bootstrap analysis to investigate the stability of the trees was performed on 1000 replicates. In the case of full length segments 5 and 6, they were processed into 2 corresponding sets of multiple sequence alignment files (ISAV-HPR0 F and ISAV-HPR0 HE gene sequences).

### Nucleotide sequence accession numbers

Sequences of segments 5 and 6 from ISAV-HPR0 cases CGA/ID758; CGA/CH1390, CGA/CH1420, CGA/CH3016, CGA/CH3201, CGA/CH3663, CGA/CH3674, CGA/CH3688; and CGA/CH1523, CGA/CH1656, CGA/CH1673, have been deposited in the GenBank database under accession numbers KF019741, KF019742; KF373252 to KF373260; and KF413748 to KF413753, respectively. Sequences of segment 6 from ISAV-HPR0 cases CGA/CH1059 to CGA/CH22377 have been deposited in the GenBank database under accession numbers KC414113 to KC414122. Sequences of segments 6 and 5 from the 2013 ISAV-HPR∆ cases CGA/3015, CGA/2826, CGA/CH1271, CGA/CH1277, CGA/2978, and CGA/CH1287 have been deposited in the GenBank database under accession numbers KF051855 to KF051934. Additional GenBank Accession numbers used in the phylogenetic analyses and the multiple alignments are shown in (Additional file 7: Table S2).

## Competing interests

The authors declare that they have no competing interests in this scientific work.

## Authors’ contributions

MGG coordinated all aspects of this study in Chile, made the veterinary investigations of the ISAV-HPR0 and the 2013 ISA outbreaks, performed sampling, the necropsy and histological analysis, coordinated the laboratory investigations, and helped to write the manuscript. MJTK performed the classic RT-PCR for ISAV segments 5 and 6 and cloned PCR products for sequencing and helped to write the manuscript. RS made the veterinary investigations of the ISAV-HPR0 and the 2013 ISA outbreaks, coordinated the laboratory investigations, and helped to write the manuscript. EL performed all the phylogenetic analyses and helped to write the manuscript. AH made the veterinary investigation of the 2013 ISAV-HPR14 case. JA isolated total RNA from tissue samples, performed all the RT-qPCR, performed the classic RT-PCR and cloned all PCR products for sequencing. DB isolated total RNA from tissue samples, performed all the RT-qPCR, performed the classic RT-PCR and cloned all PCR products for sequencing, and helped to write the manuscript. JM helped to coordinate the ISAV-HPR0 information and analysis. KOL coordinated the laboratory investigations and helped to write the manuscript. RA-H participated in manuscript preparation, review, corrections and submission. CV helped to write the manuscript. FM made the spatial and temporal analysis of ISAV-HPR0 cases and helped to write the mauscript. FSBK coordinated all viral testing and DNA sequence analysis and helped to write the manuscript. All authors read and approved the final manuscript.

## Supplementary Material

Additional file 1: Table S1 Diagnostic test results of laboratory-confirmed low pathogenic ISAV (ISAV-HPR0) and new virulent ISAV (ISAV-HPR∆) cases. Description: Table listing the detailed diagnostic information of the cases studied. Click here for file

Additional file 2: Figure S1 Phylogenetic tree showing the relationships between low virulent infectious salmon anaemia virus variants (ISAV-HPR0). Description: The analyses were performed using 1008 nucleotides of the 5′-part of the HE gene (excluding the HPR). The phylogenetic tree was constructed by maximum likelihood (ML) using Tamura-Nei and Neighbor-joining [51] as genetic distance model. The phylogeny of the ISAV-HPR0 HE shows three clusters which correspond to the three characteristic residue patterns: ^360^PAN^362^, ^360^PST^362^ and ^360^PAT^362^ in HPR, which we consider these to represent three different ISAV-HPR0 subgroups. The scale bar shows the number of nucleotide substitutions as a proportion of branch length. The identity of the ISAV virus variants and corresponding GenBank accession numbers used in the phylogenetic analyses and the multiple alignments are shown in Additional file 7: Table S2. Click here for file

Additional file 3: Table S4 Alignment of amino acid sequences in the proteolytic cleavage site of the precursor F_0_ protein from selected virulent infectious salmon anaemia virus (ISAV-HPR∆) and low pathogenic infectious salmon anaemia virus (ISAV-HPR0). Click here for file

Additional file 4: Figure S2 Phylogenetic tree showing the relationships between Chilean Infectious salmon anaemia viruses. Description: The analyses were performed using 1008 nucleotides of the 5′-part of the HE gene (excluding the HPR). The phylogenetic tree was constructed by maximum likelihood (ML) using Tamura-Nei and Neighbor-joining [51]. The ISAV-HPR0 are in blue colour while in red are the ISAV-HPR3 and ISAV-HPR14 associated to 2013 re-emergent ISA outbreak in Chile. Click here for file

Additional file 5: Figure S3 Lethargic and moribund fish in net-cage. Description: Atlantic salmon (*Salmo salar*) in vertical position at the surface of the net-cage at farm site affected by the 2013 ISA outbreak. Click here for file

Additional file 6: Figure S4 Histologic section of liver. Description: Histologic section of liver showing multifocal to diffuse acute hemorrhagic necrosis. H&E staining (100 X). The red line delimits the haemorrhagic necrosis. The left arrow show the mononuclear leukocytes cluster in hepatic parenchyma, the arrow in the middle and left arrow show mononuclear leukocytes surrounding a blood vessel. Click here for file

Additional file 7 **Additional GenBank Accession numbers used in the phylogenetic analyses and the multiple alignments.** Description: Table showing the identity of ISAV isolates and virus variants used in the phylogenetic analyses and the multiple alignments. Click here for file
