# Supplementary material for: Infectious salmon anaemia virus (ISAV) in Chilean Atlantic salmon (Salmo salar) aquaculture: emergence of low pathogenic ISAV-HPR0 and re-emergence of virulent ISAV-HPR∆: HPR3 and HPR14
Source: Virol J. 2013 Nov 23;10:344. doi: 10.1186/1743-422X-10-344 (PMC4222741; doi:10.1186/1743-422X-10-344)
Supplement: Additional file 1: Table S1 — Diagnostic test results of laboratory-confirmed low pathogenic ISAV (ISAV-HPR0) and new virulent ISAV (ISAV-HPR∆) cases. Description: Table listing the detailed diagnostic information of the cases studied. [file 1743-422X-10-344-S1.doc]

**Supplementary Table 1. Diagnostic test results of laboratory-confirmed low pathogenic ISAV (ISAV-HPR0) and new virulent ISAV (ISAV-HPRΔ) cases.**

**
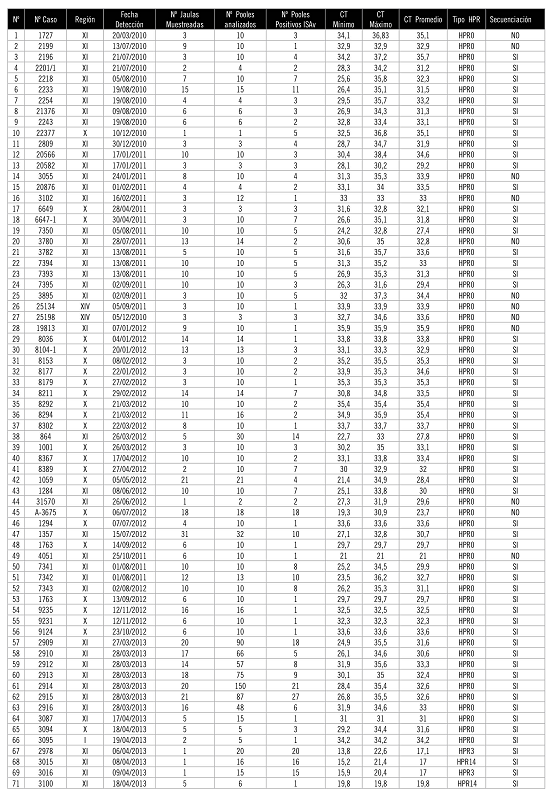
**
