# Supplementary figures and images for: Infectious salmon anaemia virus (ISAV) in Chilean Atlantic salmon (Salmo salar) aquaculture: emergence of low pathogenic ISAV-HPR0 and re-emergence of virulent ISAV-HPR∆: HPR3 and HPR14
Source: Virol J. 2013 Nov 23;10:344. doi: 10.1186/1743-422X-10-344 (PMC4222741; doi:10.1186/1743-422X-10-344)

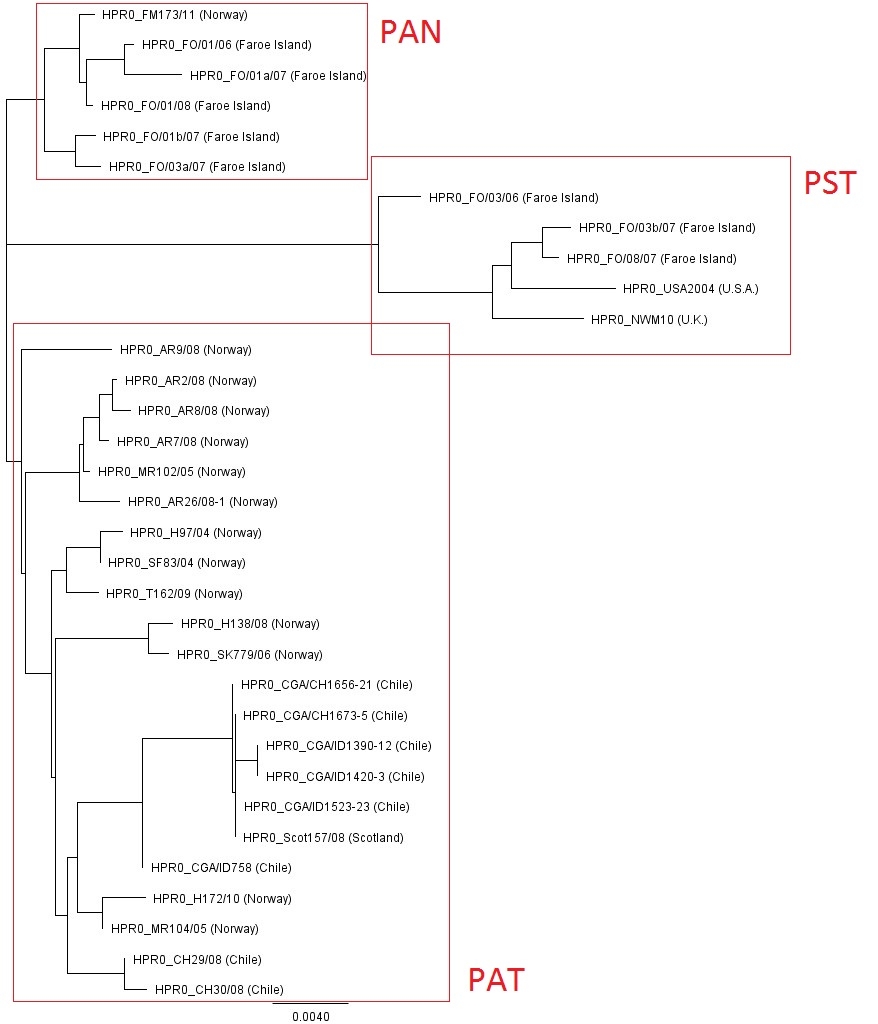

Supplement: Additional file 2: Figure S1 — Phylogenetic tree showing the relationships between low virulent infectious salmon anaemia virus variants (ISAV-HPR0). Description: The analyses were performed using 1008 nucleotides of the 5′-part of the HE gene (excluding the HPR). The phylogenetic tree was constructed by maximum likelihood (ML) using Tamura-Nei and Neighbor-joining [51] as genetic distance model. The phylogeny of the ISAV-HPR0 HE shows three clusters which correspond to the three characteristic residue patterns: 360PAN362, 360PST362 and 360PAT362 in HPR, which we consider these to represent three different ISAV-HPR0 subgroups. The scale bar shows the number of nucleotide substitutions as a proportion of branch length. The identity of the ISAV virus variants and corresponding GenBank accession numbers used in the phylogenetic analyses and the multiple alignments are shown in Additional file 7: Table S2. [file 1743-422X-10-344-S2.jpeg]

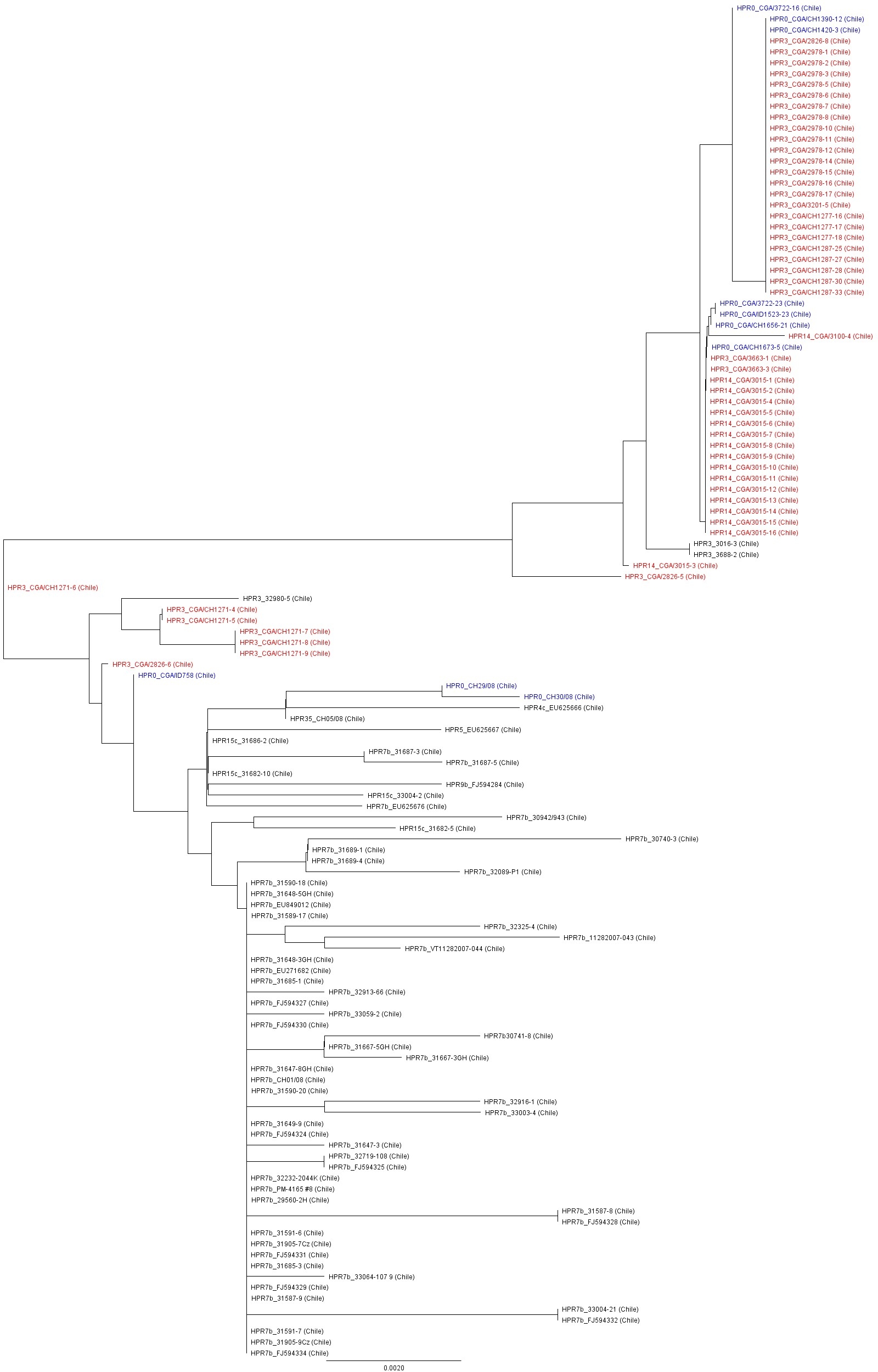

Supplement: Additional file 4: Figure S2 — Phylogenetic tree showing the relationships between Chilean Infectious salmon anaemia viruses. Description: The analyses were performed using 1008 nucleotides of the 5′-part of the HE gene (excluding the HPR). The phylogenetic tree was constructed by maximum likelihood (ML) using Tamura-Nei and Neighbor-joining [51]. The ISAV-HPR0 are in blue colour while in red are the ISAV-HPR3 and ISAV-HPR14 associated to 2013 re-emergent ISA outbreak in Chile. [file 1743-422X-10-344-S4.jpeg]

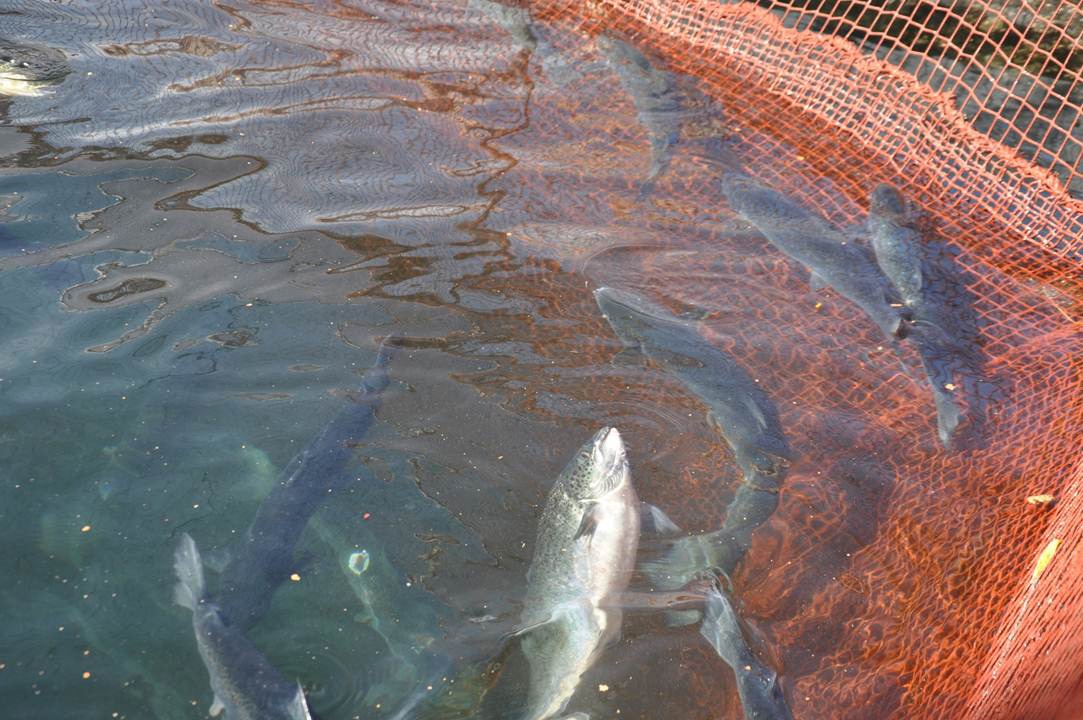

Supplement: Additional file 5: Figure S3 — Lethargic and moribund fish in net-cage. Description: Atlantic salmon (Salmo salar) in vertical position at the surface of the net-cage at farm site affected by the 2013 ISA outbreak. [file 1743-422X-10-344-S5.jpeg]

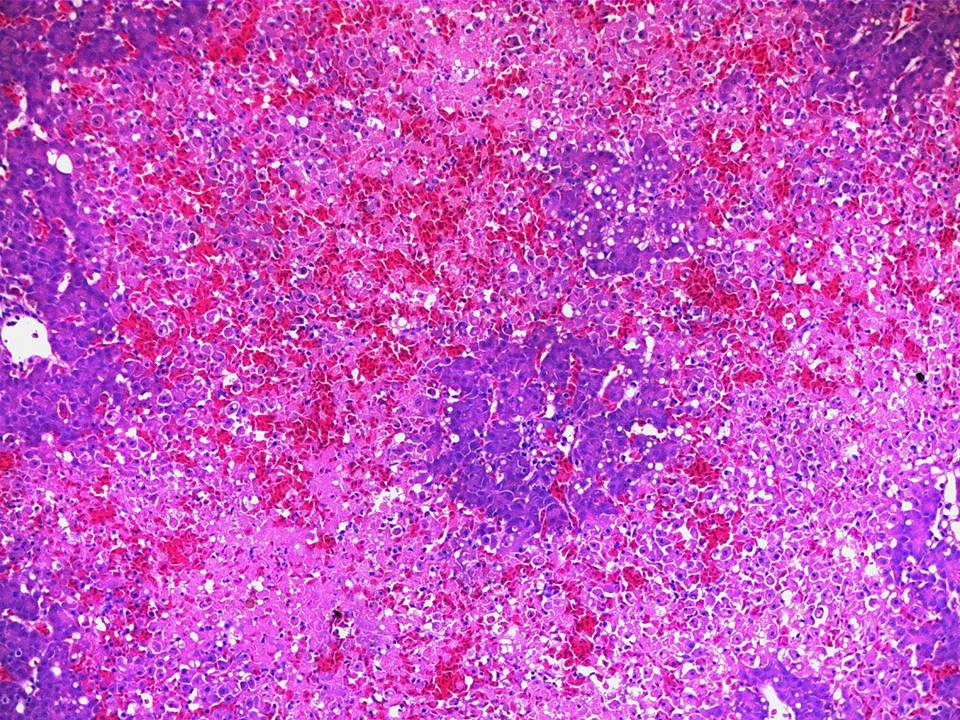

Supplement: Additional file 6: Figure S4 — Histologic section of liver. Description: Histologic section of liver showing multifocal to diffuse acute hemorrhagic necrosis. H&E staining (100 X). The red line delimits the haemorrhagic necrosis. The left arrow show the mononuclear leukocytes cluster in hepatic parenchyma, the arrow in the middle and left arrow show mononuclear leukocytes surrounding a blood vessel. [file 1743-422X-10-344-S6.jpeg]
