# Supplementary material for: Infectious salmon anaemia virus (ISAV) in Chilean Atlantic salmon (Salmo salar) aquaculture: emergence of low pathogenic ISAV-HPR0 and re-emergence of virulent ISAV-HPR∆: HPR3 and HPR14
Source: Virol J. 2013 Nov 23;10:344. doi: 10.1186/1743-422X-10-344 (PMC4222741; doi:10.1186/1743-422X-10-344)
Supplement: Additional file 3: Table S4 — Alignment of amino acid sequences in the proteolytic cleavage site of the precursor F0 protein from selected virulent infectious salmon anaemia virus (ISAV-HPR∆) and low pathogenic infectious salmon anaemia virus (ISAV-HPR0). [file 1743-422X-10-344-S3.doc]

**Table S4. Alignment of amino acid sequences in the proteolytic cleavage site of the precursor F_0_ protein from selected virulent infectious salmon anaemia virus (ISAV-HPRΔ) and low pathogenic infectious salmon anaemia virus (ISAV-HPR0)**

**
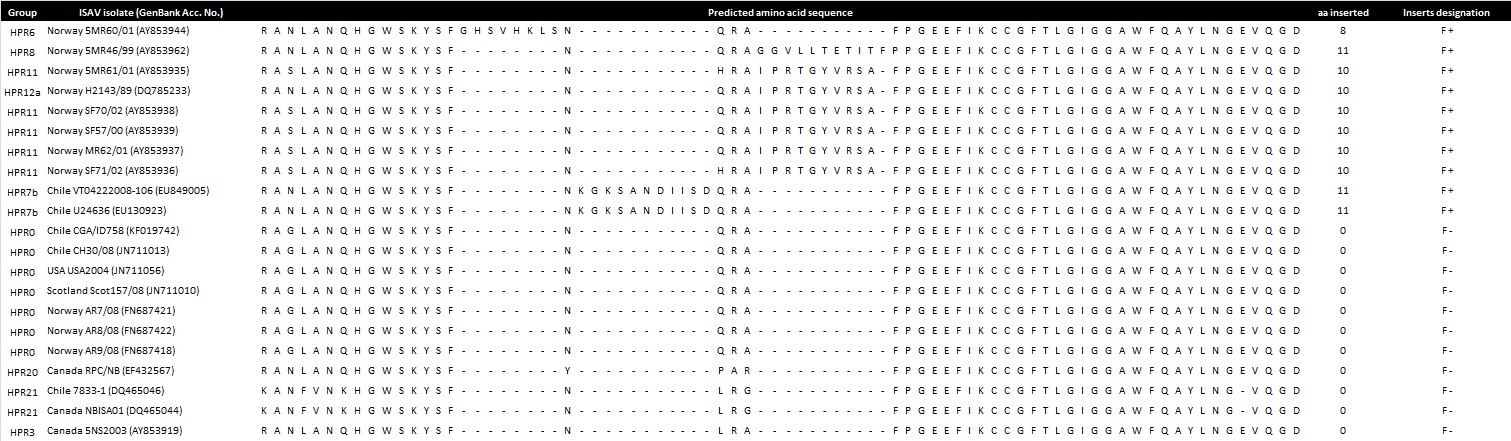
**
