# Supplementary material for: Infectious salmon anaemia virus (ISAV) in Chilean Atlantic salmon (Salmo salar) aquaculture: emergence of low pathogenic ISAV-HPR0 and re-emergence of virulent ISAV-HPR∆: HPR3 and HPR14
Source: Virol J. 2013 Nov 23;10:344. doi: 10.1186/1743-422X-10-344 (PMC4222741; doi:10.1186/1743-422X-10-344)
Supplement: Additional file 7 — Additional GenBank Accession numbers used in the phylogenetic analyses and the multiple alignments. Description: Table showing the identity of ISAV isolates and virus variants used in the phylogenetic analyses and the multiple alignments. [file 1743-422X-10-344-S7.doc]

**Supplementary Table 2.** Additional GenBank Accession numbers used in the phylogenetic analyses and the multiple alignments

| **Group** | **Isolate** | **Segment 5** | **Segment 6** | **Reference** | **Year** |
| --- | --- | --- | --- | --- | --- |
| HPRO | AR7/08 (Norway) | FN687421 | FN687351 | Lyngstad *et al*. [18] | 2008 |
| HPRO | AR8/08 (Norway) | FN687422 | FN687352 | Lyngstad *et al*. [18] | 2008 |
| HPRO | AR9/08 (Norway) | FN687423 | FN687353 | Lyngstad *et al*. [18] | 2008 |
| HPRO | AR26/08-1 (Norway) | FN687425 | FN687356 | Lyngstad *et al*. [18] | 2008 |
| HPRO | CH29/08 (Chile) | JN711012 | JN711094 | Plarre *et al.* [21] | 2008 |
| HPRO | CH30/08 (Chile) | JN711013 | JN711095 | Plarre *et al.* [21] | 2008 |
| HPRO | FM173/11 (Norway) | JN711019 | JN711060 | Plarre *et al.* [21] | 2011 |
| HPRO | FO/01/06 (Faroe Island) | - | HQ664992 | Christiansen *et al.* [19] | 2006 |
| HPRO | FO/01/08 (Faroe Island) | - | HQ664999 | Christiansen *et al.* [19] | 2006 |
| HPRO | FO/01a/07 (Faroe Island) | - | HQ664994 | Christiansen *et al.* [19] | 2006 |
| HPRO | FO/01b/07 (Faroe Island) | - | HQ664995 | Christiansen *et al.* [19] | 2006 |
| HPRO | FO/03/06 (Faroe Island) | - | HQ664993 | Christiansen *et al.* [19] | 2006 |
| HPRO | FO/03a/07 (Faroe Island) | - | HQ664996 | Christiansen *et al.* [19] | 2006 |
| HPRO | FO/03b/07 (Faroe Island) | - | HQ664997 | Christiansen *et al.* [19] | 2006 |
| HPRO | FO/08/07 (Faroe Island) | - | HQ664998 | Christiansen *et al.* [19] | 2006 |
| HPRO | H97/04 (Norway) | JN711024 | DQ108604 | Nylund *et al.* [14] | 2004 |
| HPRO | H138/08 (Norway) | JN711022 | JN711063 | Plarre *et al.* [21] | 2008 |
| HPRO | H172/10 (Norway) | JN711023 | JN711064 | Plarre *et al.* [21] | 2010 |
| HPRO | MR102/05 (Norway) | EU851044 | DQ108605 | Vike *et al.* [23] | 2005 |
| HPRO | MR104/05 (Norway) | JN711026 | DQ108607 | Nylund *et al.* [14] | 2004 |
| HPRO | NWM10(U.K.) | - | FJ178189 | McBeath *et al.* [16] | 2007 |
| HPRO | Scot157/08 (Scotland) | JN711010 | JN711096 | Plarre *et al*. [21] | 2008 |
| HPRO | SF83/04 (Norway) | AY744392 | AY973190 | Nylund *et al*. [14] | 2004 |
| HPRO | SK779/06 (Norway) | EU118819 | EU118820 | Markussen *et al*. [15] | 2006 |
| HPRO | T162/09 (Norway) | JN711053 | JN711091 | Plarre *et al.* [21] | 2009 |
| HPRO | USA2004 (USA) | JN711056 | AY973194 | Nylund *et al.* [14] | 2004 |
| HPR1 | FM86/04 (Norway) | - | AY971659 | Devold *et al.* [52] | 2004 |
| HPR1 | 5H1/87 (Norway) | AY853942 | - | Devold *et al.* [52] | 1987 |
| HPR2 | 6/91 (Norway) | - | AF364894 | Devold *et al.* [53] | 1991 |
| HPR2 | 38/98 (Norway) | - | AF364874 | Devold *et al*. [53] | 1998 |
| HPR2 | 1490/98(Scotland) | - | AF391126 | Kibenge *et al.* [54] | 1998 |
| HPR2 | AF220607 (Norway) | - | AF220607 | Rimstad *et al.* [4] | 2001 |
| HPR2 | NT141/08 (Norway) | JN711031 | JN711070 | Plarre *et al.* [21] | 2008 |
| HPR2 | 5T33/98 (Norway) | AY853969 | - | Devold *et al.* [52] | 1998 |
| HPR3 | 32980-5 (Chile) | - | FJ594294 | Kibenge *et al.* [8] | 2008 |
| HPR3 | F2002 (Faroe Island) | - | AF526263 | Plarre *et al.* [21] | 2003 |
| HPR3 | NT115/05 (Norway) | JN711029 | JN711068 | Christiansen *et al.* [19] | 2005 |
| HPR3 | ST110/05 (Norway) | JN711034 | DQ108598 | Nylund *et al.* [14] | 2005 |
| HPR3 | T91/04 (Norway) | - | AY971667 | Devold *et al.* [52] | 2004 |
| HPR3 | U5575-1 (Canada) | EF217314 | AF294881 | Kibenge *et al.* [28] | 2005 |
| HPR3 | FM14/05 (Norway) | JN711016 | JN711057 | Plarre *et al.* [21] | 2005 |
| HPR4 | Gullesfjord/94 (Norway) | - | AF302801 | Krossøy *et al.* [5] | 1994 |
| HPR4 | 5T10/93 (Norway) | AY853922 | - | Devold *et al.* [52] | 1993 |
| HPR4c | VT1128007-35 (Chile) | - | EU625666 | Kibenge *et al.* [8] | 2008 |
| HPR5 | 5SF14/95 (Norway) | AY853925 | - | Devold *et al.* [52] | 1995 |
| HPR5 | VT11282007-38 (Chile) | - | EU625667 | Kibenge *et al.* [8] | 2008 |
| HPR5 | T122/07 (Norway) | JN711037 | JN711075 | Plarre *et al.* [21] | 2007 |
| HPR5 | T129/07 (Norway) | JN711039 | JN711077 | Plarre *et al.* [21] | 2007 |
| HPR5 | EU849008 (Chile) | EU849008 | EU625667 | Kibenge *et al.* [8] | 2007 |
| HPR5 | T145/08 (Norway) | JN711043 | JN711081 | Plarre *et al.* [21] | 2008 |
| HPR6 | 5ST25/97 (Norway) | AY853926 | AF364885 | Devold *et al.* [53] | 1997 |
| HPR6 | 5ST27/97 (Norway) | AY853929 | AF364897 | Devold *et al.* [53] | 1997 |
| HPR6 | 5MR60/01 (Norway) | AY853944 | AY127876 | Devold *et al.* [53] | 2001 |
| HPR7b | 30741-8 (Chile) | FJ592150 | FJ594306 | Kibenge *et al.* [8] | 2008 |
| HPR7b | 5N29/97 (Norway) | AY853920 | AF364872 | Devold *et al.* [53] | 1997 |
| HPR7b | 29560-2H (Chile) | - | FJ594303 | Kibenge *et al.* [8] | 2008 |
| HPR7b | 30740-3 (Chile) | FJ592143 | FJ594297 | Kibenge *et al.* [8] | 2008 |
| HPR7b | 30942/943 (Chile) | FJ592140 | FJ594292 | Kibenge *et al.* [8] | 2008 |
| HPR7b | 31587-8 (Chile) | FJ592165 | FJ594328 | Kibenge *et al.* [8] | 2008 |
| HPR7b | 31587-9 (Chile) | - | FJ594330 | Kibenge *et al.* [8] | 2008 |
| HPR7b | 31587-17 (Chile) | - | FJ594317 | Kibenge *et al.* [8] | 2008 |
| HPR7b | 31589-18 (Chile) | FJ592166 | FJ594329 | Kibenge *et al.* [8] | 2008 |
| HPR7b | 31590-20 (Chile) | FJ592155 | FJ594312 | Kibenge *et al.* [8] | 2008 |
| HPR7b | 31590-6 (Chile) | FJ592167 | FJ594331 | Kibenge *et al.* [8] | 2008 |
| HPR7b | 31591-7 (Chile) | FJ592159 | FJ594320 | Kibenge *et al.* [8] | 2008 |
| HPR7b | 31647-3 (Chile) | FJ592154 | FJ594311 | Kibenge *et al.* [8] | 2008 |
| HPR7b | 31647-8GH (Chile) | FJ592152 | FJ594308 | Kibenge *et al.* [8] | 2008 |
| HPR7b | 31648-3GH (Chile) | FJ592168 | FJ594334 | Kibenge *et al.* [8] | 2008 |
| HPR7b | 31648-5GH (Chile) | FJ592158 | FJ594318 | Kibenge *et al.* [8] | 2008 |
| HPR7b | 31649-9 (Chile) | - | FJ594324 | Kibenge *et al.* [8] | 2008 |
| HPR7b | 31667-3GH (Chile) | FJ592149 | FJ594305 | Kibenge *et al.* [8] | 2008 |
| HPR7b | 31667-5GH (Chile) | FJ592151 | FJ594307 | Kibenge *et al.* [8] | 2008 |
| HPR7b | 31685-1 (Chile) | FJ592164 | FJ594327 | Kibenge *et al.* [8] | 2008 |
| HPR7b | 31685-3 (Chile) | FJ592153 | FJ594310 | Kibenge *et al.* [8] | 2008 |
| HPR7b | 31687-3 (Chile) | FJ592136 | FJ594286 | Kibenge *et al.* [8] | 2008 |
| HPR7b | 31689-5 (Chile) | FJ592137 | FJ594287 | Kibenge *et al.* [8] | 2008 |
| HPR7b | 31689-1 (Chile) | FJ592144 | FJ594299 | Kibenge *et al.* [8] | 2008 |
| HPR7b | 31689-4 (Chile) | FJ592145 | FJ594298 | Kibenge *et al.* [8] | 2008 |
| HPR7b | 31790-3GH (Chile) | FJ592148 | FJ594304 | Kibenge *et al.* [8] | 2008 |
| HPR7b | 31790-9GH (Chile) | FJ592147 | FJ594302 | Kibenge *et al.* [8] | 2008 |
| HPR7b | 31905-7Cz (Chile) | FJ592160 | FJ594321 | Kibenge *et al.* [8] | 2008 |
| HPR7b | 31905-9Cz (Chile) | FJ592156 | FJ594313 | Kibenge *et al.* [8] | 2008 |
| HPR7b | 32089-P1 (Chile) | FJ592146 | FJ594301 | Kibenge *et al.* [8] | 2008 |
| HPR7b | 32232-2032LK (Chile) | FJ592161 | FJ594322 | Kibenge *et al.* [8] | 2008 |
| HPR7b | 32232-2044K (Chile) | - | FJ594314 | Kibenge *et al.* [8] | 2008 |
| HPR7b | 32325-4 (Chile) | - | FJ594296 | Kibenge *et al.* [8] | 2008 |
| HPR7b | 32719-108 (Chile) | - | FJ594325 | Kibenge *et al.* [8] | 2008 |
| HPR7b | 32913-66 (Chile) | FJ592162 | FJ594323 | Kibenge *et al.* [8] | 2008 |
| HPR7b | 32916-1 (Chile) | - | FJ594315 | Kibenge *et al.* [8] | 2008 |
| HPR7b | 33003-4 (Chile) | - | FJ594316 | Kibenge *et al.* [8] | 2008 |
| HPR7b | 33004-21 (Chile) | - | FJ594332 | Kibenge *et al.* [8] | 2008 |
| HPR7b | 33059-2(Chile) | - | FJ594309 | Kibenge *et al.* [8] | 2008 |
| HPR7b | 04222008-106 (Chile) | EU849005 | EU849012 | Kibenge *et al.* [8] | 2008 |
| HPR7b | 04222008-107(Chile) | EU849007 | EU849013 | Kibenge *et al.* [8] | 2008 |
| HPR7b | 11282007-042 (Chile) | - | EU625679 | Kibenge *et al.* [8] | 2007 |
| HPR7b | 11282007-043 (Chile) | - | EU625680 | Kibenge *et al.* [8] | 2007 |
| HPR7b | CH01/08 (Chile) | EU851042 | EU851043 | Vike *et al.* [23] | 2008 |
| HPR7b | EU271682 (Chile) | EU449765 | EU271682 | Kibenge *et al.* [8] | 2007 |
| HPR7b | EU625676 (Chile) | EU849006 | EU625676 | Kibenge *et al.* [8] | 2007 |
| HPR7b | EU849012 (Chile) | EU849005 | EU849012 | Kibenge *et al.* [8] | 2008 |
| HPR7b | FJ594324 (Chile) | - | FJ594324 | Kibenge *et al.* [8] | 2008 |
| HPR7b | FJ594325 (Chile) | - | FJ594325 | Kibenge *et al.* [8] | 2008 |
| HPR7b | FJ594327 (Chile) | FJ592164 | FJ594327 | Kibenge *et al.* [8] | 2008 |
| HPR7b | FJ594328 (Chile) | FJ592165 | FJ594328 | Kibenge *et al.* [8] | 2008 |
| HPR7b | FJ594329 (Chile) | FJ529166 | FJ594329 | Kibenge *et al.* [8] | 2008 |
| HPR7b | FJ594330 (Chile) | - | FJ594330 | Kibenge *et al.* [8] | 2008 |
| HPR7b | FJ594331 (Chile) | FJ592167 | FJ594331 | Kibenge *et al.* [8] | 2008 |
| HPR7b | FJ594332 (Chile) | - | FJ594332 | Kibenge *et al.* [8] | 2008 |
| HPR7b | FJ594334 (Chile) | FJ592168 | FJ594334 | Kibenge *et al.* [8] | 2008 |
| HPR7b | LochNevis/98 (Scotland) | - | AF302802 | Krossøy *et al.* [5] | 1998 |
| HPR7b | NT81/03 (Norway) | - | AY973184 | Nylund *et al.* [14] | 2003 |
| HPR7b | PM-4165 #8 (Chile) | FJ592163 | FJ594326 | Kibenge *et al.* [8] | 2008 |
| HPR7b | VT11282007-044 (Chile) | - | EU625681 | Kibenge *et al.* [8] | 2008 |
| HPR7b | 5N32/98 (Norway) | AY853921 | - | Devold *et al.* [52] | 1998 |
| HPR8 | 46/99 (Norway) | - | AF364896 | Devold *et al.* [52] | 1999 |

| HPR8 | 48/99 (Norway) | - | AF364878 | Devold *et al.* [52] | 1999 |
| --- | --- | --- | --- | --- | --- |
| HPR8 | 5MR46/99 | AY853962 | - | Devold *et al.* [52] | 1999 |
| HPR9 | 47/99 (Norway) | - | AF364888 | Devold *et al.* [52] | 1999 |
| HPR9 | FM116/08 (Norway) | JN711017 | JN711058 | Plarre *et al.* [21] | 2006 |
| HPR9b | FJ594284 (Chile) | FJ592134 | FJ594284 | Kibenge *et al.* [8] | 2008 |
| HPR10 | 52/00 (Norway) | - | AF364892 | Devold *et al.* [52] | 2000 |
| HPR10 | H93/04 (Norway) | - | AY973179 | Nylund *et al.* [14] | 2004 |
| HPR11 | 54/00 (Norway) | - | AF364884 | Devold *et al.* [53] | 2000 |
| HPR11 | 5MR71/02 (Norway) | AY853936 | - | Devold *et al.* [52] | 2002 |
| HPR11 | ST61/01 (Norway) | - | AY127877 | Nylund *et al.* [13] | 2001 |
| HPR11 | SF70/02 (Norway) | AY853938 | AY127880 | Devold *et al.* [52] | 2002 |
| HPR11 | SF57/00 (Norway) | AY853939 | - | Devold *et al.* [52] | 2000 |
| HPR12 | N5/89 (Norway) | - | AY127882 | Nylund *et al.* [14] | 1998 |
| HPR12a | H2143 (Norway) | DQ785233 | DQ785247 | Markussen *et al.* [25] | 1989 |
| HPR14 | 21/96 (Norway) | - | AF364886 | Devold *et al.* [53] | 1996 |
| HPR14 | FM106/05 (Norway) | JN711014 | DQ108599 | Nylund *et al.* [14] | 2005 |
| HPR14 | Veday/99 (Norway) | - | AF302803 | Krossøy *et al.* [5] | 1999 |
| HPR15 | 810/99 (Norway) | EF217313 | AF378180 | Kibenge et al. [28] | 1999 |
| HPR15 | Bremnes/98 (Norway) | - | AF302799 | Krossøy *et al.* [5] | 1998 |
| HPR15c | 31682-5 (Chile) | FJ592141 | FJ594293 | Kibenge *et al.* [8] | 2008 |
| HPR15c | 31682-10 (Chile) | FJ592133 | FJ594283 | Kibenge *et al.* [8] | 2008 |
| HPR15c | 31682-2 (Chile) | - | FJ594288 | Kibenge *et al.* [8] | 2008 |
| HPR15c | 33004-2 (Chile) | - | FJ594285 | Kibenge *et al.* [8] | 2008 |
| HPR16 | T90/04 (Norway) | JN711055 | AY971666 | Nylund *et al.* [14] | 2004 |
| HPR17 | MR 103/05 (Norway) | JN711025 | DQ108606 | Nylund *et al.* [14] | 2005 |
| HPR19 | 18/96 (Norway) | - | AF364869 | Devold *et al.* [53] | 1996 |
| HPR 31 | T121/07 (Norway) | JN711036 | JN711074 | Plarre *et al.* [21] | 2007 |
| HPR31 | T126/07 (Norway) | JN711038 | JN711076 | Plarre *et al.* [21] | 2007 |
| HPR33 | N127a/07 (Norway) | - | JN71166 | Plarre *et al.* [21] | 2007 |
| HPR34 | NT134/08 (Norway) | JN71130 | JN71169 | Plarre *et al.* [21] | 2008 |
| HPR35 | CH05/08 (Chile) | JN711011 | JN71193 | Plarre *et al.* [21] | 2008 |
| HPR35 | MR 139/08 (Norway) | JN711027 | JN711065 | Plarre *et al.* [21] | 2008 |
| HPR35 | ST143/08 (Norway) | JN711035 | JN11073 | Plarre *et al.* [21] | 2008 |
| HPR36 | Vir22 (Norway) | DQ785244 | DQ785258 | Markussen *et al.* [25] | 2006 |
| HPR36 | Vir25 (Norway) | DQ785242 | DQ785256 | Markussen *et al.* [25] | 2006 |

52. Devold M, Karlsen M, Nylund A: **Sequence analysis of the fusion protein gene from infectious salmon anemia virus isolates: evidence of recombination and reassortment.** *J Gen Virol* 2006, **87:**2031–2040.

53. Devold M, Falk K, Dale OB, Krossøy B, Biering E, Aspehaug V, Nilsen F, Nylund A: **Strain variation, based on the hemagglutinin gene, in Norwegian ISA virus isolates collected from 1987 to 2001: indications of recombination.** *Dis Aquat Org* 2001, **47:**119–128.

54. Kibenge FSB, Kibenge MJT, McKenna PK, Stothard P, Marshall R, Cusack RR, McGeachy S: **Antigenic variation among isolates of infectious salmon anaemia virus (ISAV) correlates with genetic variation of the viral haemagglutinin gene.** *J Gen Virol* 2001, **82:**2869–2879.
